# Anodic Activation of Prussian Blue Analog Leads to Highly Active Cobalt‐Doped Nickel (Oxy)Hydroxide for Organic Oxidation Reactions

**DOI:** 10.1002/chem.202404174

**Published:** 2025-01-13

**Authors:** Toufik Ansari, Debabrata Bagchi, Suptish Ghosh, Jan Niklas Hausmann, Arindam Indra, Prashanth W. Menezes

**Affiliations:** ^1^ Department of Chemistry IIT (BHU) Varanasi UP-221005 India; ^2^ Material Chemistry Group for Thin Film Catalysis CatLab Helmholtz-Zentrum Berlin für Materialien und Energie, Albert-Einstein-Str. 15 12489 Berlin Germany; ^3^ Department of Chemistry Metalorganics and Inorganic Materials Technische Universität Berlin, Straße des 17 Juni 115, Sekr. C2 10623 Berlin Germany

**Keywords:** Prussian blue analog (PBA), Metal oxyhydroxides, Electrochemical deposition, Nanosheet structure, Benzyl alcohol oxidation

## Abstract

Water‐assisted electrocatalytic oxidation of alcohols into valuable chemicals is a promising strategy to circumvent the sluggish kinetics of water oxidation, while also reducing cell voltage and improving energy efficiency. Recently, transition metal (TM)‐based catalysts have been investigated for anodic alcohol oxidation, but success has been limited due to competition from the oxygen evolution reaction (OER) within the working regime. In this study, NiCo‐based Prussian blue analog (PBA) was electrochemically activated at the anodic potential to produce a Co−Ni(O)OH active catalyst with a nanosheet‐like architecture. This catalyst was further employed for the selective oxidation of benzyl alcohol (PhCH_2_OH) to benzoic acid (PhCOOH), achieving a 97 % Faradaic efficiency (FE). The electrochemical activity of Co−Ni(O)OH was also compared with hydrothermally prepared CoNi‐LDH, demonstrating that the PBA‐derived Co−Ni(O)OH was more effective.

## Graphical abstract

Anodic activation of NiCo Prussian blue analog yields a highly active Co‐doped Ni(O)OH catalyst with nanosheet architecture, enabling efficient and selective oxidation of benzyl alcohol. This catalyst achieves up to 97 % Faradaic efficiency for the production of benzoic acid and industrial‐level current densities at low applied potential, outperforming conventional pristine Ni and Co‐based analogs.

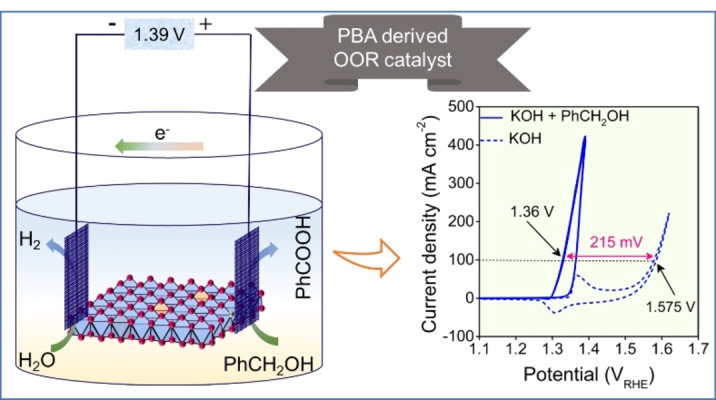

## 1 Introduction

Electrocatalysis in water is considered a green approach for organic synthesis as it only requires renewable energy and water, which are environmentally friendly.^[1]^ Over the past few decades, electrocatalysis has been explored to drive a wide range of essential organic redox reactions.^[2]^ On the other hand, the production of green hydrogen by water electrolysis has attained immense interest for sustainable energy conversion. However, the production of H_2_ by electrocatalytic water splitting suffers from challenges like (i) thermodynamically uphill and kinetically sluggish OER at the anode‐reducing the overall efficiency of the cell, (ii) high cell voltage, (iii) poor energy efficiency, (iv) the mixing of H_2_ and O_2_ to form an explosive mixture.[^3^, ^4^, ^5^, ^6^, ^7^]

The combination of anodic alcohol oxidation reaction (AOR) with cathodic H_2_ evolution can significantly reduce the electricity consumption of the cell with the production of value‐added product(s).^[8]^ However, AOR in the aqueous medium suffers from the challenges of low Faradaic efficiency (FE) because of the competition from the OER. Although the thermodynamic potential for PhCH_2_OH oxidation 0.48 V versus reversible hydrogen electrode (vs. RHE) is significantly lower than that of water oxidation (1.23 V vs. RHE), in practice, the anodic oxidation of the former takes place at a high potential (>1.4 V vs. RHE).[^9^, ^10^, ^11^] This leads to competition from the OER, reducing the FE of AOR. To improve the FE, AOR was often carried out at a low current density, which limits its practical applications with poor energy efficiency and high cost of the value‐added products. Therefore, it is crucial to develop a suitable catalyst that can achieve AOR with an industrial‐scale current density (400 mA cm^−2^) at a potential lower than that of the OER onset.[^12^, ^13^, ^14^, ^15^, ^16^]

In recent years, metal‐oxyhydroxides (M(O)OH) have gained tremendous attention as an electrocatalyst because of their high efficiency in AOR.[^17^, ^18^, ^19^] The outstanding activity of the M(O)OH can be attributed to its high active surface area and a large number of surface‐exposed active sites, which collectively contribute to its catalytic performance. Further, the manipulation of the electronic structure and local atomic structure was found to be crucial to varying the electrochemical performance of M(O)OH.[^2^, ^3^, ^9^, ^20^, ^21^, ^22^]

In most cases, M(O)OH with a single metal ion exhibits poor AOR activity.[^23^, ^24^, ^25^, ^26^, ^27^] However, the structure modulation by the introduction of hetero atoms, heterojunction formation, structural defects, cationic and anionic vacancies, etc. were explored to tune the properties of M(O)OH, ultimately enhancing its AOR activity.^[28]^ For example, Li et al. reported Au/CoOOH for the anodic oxidation of PhCH_2_OH at an industrial‐scale current density.^[26]^ Although Co(O)OH can oxidize PhCH_2_OH at a low potential, the poor adsorption of PhCH_2_OH on the catalyst surface results in poor activity. However, the interface between Au and Co(O)OH facilitates the adsorption of the substrate on the catalyst surface, improving the catalytic activity.[^26^, ^29^, ^30^] The introduction of different 3d‐metal ions (Cu, Zn, and Ni) in the structure of Co(O)OH and its effect on the electrocatalytic oxidation of PhCH_2_OH oxidation was explored by Lei et al.[^31^, ^32^] The DFT study revealed that the overlap of d‐orbitals of Cu and Co led to a shift of Co's d‐orbital towards the Fermi level to increase the adsorption energy of PhCH_2_OH, thereby, enhancing catalytic efficiency of Cu‐Co(O)OH.

Similarly, various metal‐doped Ni(O)OH have been reported for the AOR. For example, Wei et al. used Fe−Ni(O)OH for PhCH₂OH oxidation and found a significant relationship between the redox peak positions of Ni^2+/3+^ and PhCH₂OH oxidation activity.[^33^, ^34^, ^35^, ^36^] Their mechanistic studies demonstrate that Ni^3+^ plays a crucial role in PhCH₂OH oxidation.

To achieve an industrial‐scale current density, Hausmann et al. utilized plasma‐treated nickel foam (NF) to enhance the number of active sites for PhCH_2_OH oxidation. Their catalyst exhibited exceptional activity, achieving over 800 mA cm^−2^ current density.[^34^, ^37^] Xianlang et al. demonstrated the importance of defects in the catalyst structure for PhCH_2_OH oxidation.^[38]^ The leaching of Zn from Zn−Ni(OH)_2_ produced a cationic vacancy‐rich catalyst, resulting in a significant improvement in the catalytic activity. Our study also showed that the electronic structure of Fe−Co(O)OH nanosheets, prepared by different methods, had a crucial effect in controlling the AOR activity.[^34^, ^37^]

Importantly, Ni has been recognized as a common metal ion in Fe‐based and Co‐based catalyst systems used for organic oxidation reactions.^[39]^ For example, Liu et al. synthesized Co_0.83_Ni_0.17_/AC and demonstrated that the presence of metallic cobalt significantly enhances the material's conductivity, while nickel offers the active sites for the oxidation of PhCH_2_OH.^[40]^ Xu et al. revealed that Fe‐based layered double hydroxides (LDHs) can modulate the overlap of the d‐band in LDHs, optimize the local active structure, and thereby enhance the electrocatalytic oxidation of PhCH_2_OH.^[41]^ Huang et al. developed Fe/Co(oxide) heterostructures to regulate surface structural properties for improved PhCH_2_OH oxidation. Their findings indicate that interface engineering and the heterostructure formation tunes the local crystallinity and defects, that enhance the PhCH_2_OH oxidation activity. In particular, the defective sites concentrated at the interfaces promote the adsorption and dissociation of intermediates during the electrocatalytic process.^[42]^ Similarly, Li et al. synthesized 2D Ni‐based CC@NiO/Ni_3_S_2_ through a simple one‐step electrodeposition method.^[43]^ The NiO/Ni_3_S_2_ heterointerfaces exposed more active sites, enhanced mass and charge diffusion, and provided unique interfacial interactions that facilitate charge redistribution, thereby promoting the formation of crucial reaction intermediates. Consequently, the CC@NiO/Ni_3_S_2_ demonstrated superior electrochemical activity with excellent FE.

**Figure 1:**
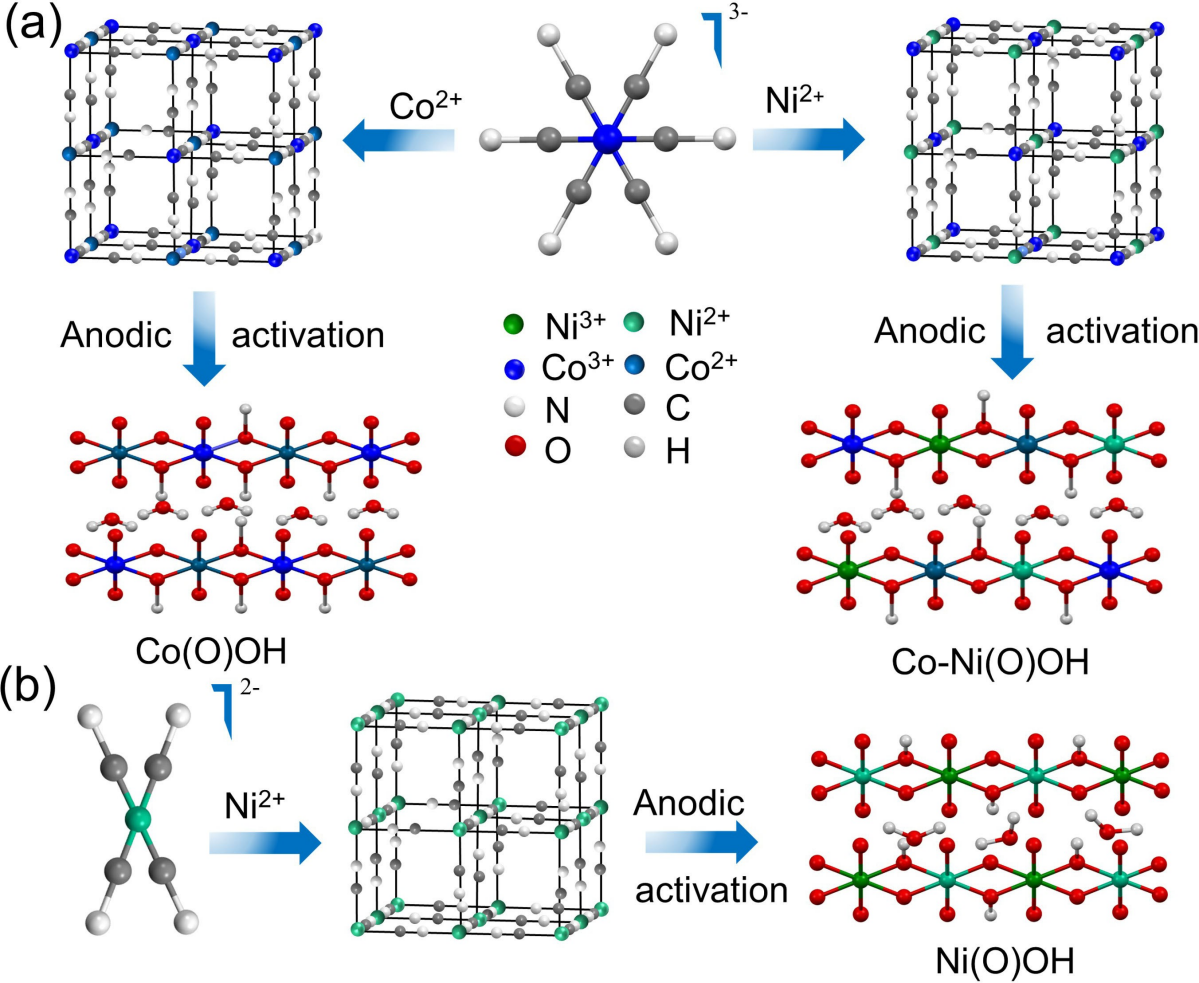
(a and b) Schematic illustration showing the formation of Co(O)OH, CoNi(O)OH and Ni(O)OH by the anodic activation of prussian blue analogs (PBAs) and NiNi‐coordination polymer (CP).

The above studies prompted us to synthesize Co‐NiOOH nanosheets by different methods and evaluate their electro‐catalytic activity for the oxidation of PhCH_2_OH to PhCOOH. For this purpose, we have used NiCo‐PBA, CoCo‐PBA, and NiNi‐CP (CP = coordination polymer) as the precatalysts and produced active catalysts Co−Ni(O)OH, Co(O)OH, and Ni(O)OH by electrochemical anodic activation (named as CoNi−A, Co−A, and Ni−A, respectively) (Figure 1). The PBA‐derived Co−Ni(O)OH nanosheets (CoNi−A) with a tuned electronic structure showed superior AOR, achieving a complete conversion of PhCH_2_OH to PhCOOH at 1.39 V against RHE with ≥97 % FE. Furthermore, an industrial current density of 400 mA cm^−2^ was reached at a very low electrode potential of 1.38 V vs. RHE.

## 2 Results and Discussion

### 2.1 Synthesis and Characterization of the Precatalysts

The precatalysts NiCo‐PBA, CoCo‐PBA, and NiNi‐CP were synthesized by a co‐precipitation method. Furthermore, powder X‐ray diffraction (PXRD) confirmed the formation of the cubic phase of NiCo‐PBA, and CoCo‐PBA (Ni_3_[Co(CN)_6_]_2_⋅12H_2_O, PDF#89–3738; space group: *F*‐43 *m*, a=b=c=10.14 Å, *α*=*β*=*γ*=90°) (Figure S1).^[13]^ Additionally, all the PXRD diffraction peaks in NiNi‐CP were attributed to the Hofmann‐type Ni(H_2_O)_2_[Ni(CN)_4_]⋅xH_2_O, which matches well with the previous reports.[^44^, ^45^]

In CoCo‐PBA, the cationic site is occupied by Co^2+^, whereas in NiCo‐PBA, the cationic site is occupied by Ni^2+^. Since the ionic radius of Ni^2+^ is smaller than that of Co^2+^, this leads to an increase in the electronegativity of nitrogen in NiCo‐PBA compared to CoCo‐PBA.[^46^, ^47^] As a result, the bond strength of the −C≡N bond increases, leading to greater lattice contraction and a positive shift in the PXRD patterns (2θ=0.3–0.4) of NiCo‐PBA compared to CoCo‐PBA (Figure S1).[^46^, ^47^] The Fourier‐transformed infrared (FTIR) spectra showed an intense peak for the asymmetric stretching vibration of the bridged –CN group of the precatalysts. For NiCo‐PBA, a positive shift of the −CN peak (18 cm^−1^) compared to CoCo‐PBA was observed because of the enhanced −C≡N bond strength (Figure S2).[^46^, ^48^] To gain insight into the morphology of the precatalyst, SEM (scanning electron microscopy) and TEM (transmission electron microscopy) analyses were performed. SEM and TEM images reveal an irregular shape for the NiCo‐PBA particles. The SEM‐EDX mapping revealed a uniform distribution of the elements C, N, O, Co, and Ni. (Figure S3–S4).

### 2.2 Electrochemical Activation of the Precatalyst

Both PBAs and NiNi‐CP were electrophoretically deposited (EPD) on NF to obtain binder‐free catalyst films on the NF substrates (see details in SI).^[49]^ The electrochemical activation of the precatalysts (PBAs) was carried out in 1.0 M KOH solution using cyclic voltammetry (CV) in a standard three‐electrode system. The CV scans were performed in the range of 1.0–2.0 V vs. RHE at a scan rate of 5 mV/s. The electrochemical activation of NiCo‐PBA, CoCo‐PBA, and NiNi‐CP formed CoNi−A [CoNi(O)OH], Co−A [Co(O)OH/Co(OH)_2_], and Ni−A [Ni(O)OH] (see later).

### 2.3 Characterization of the Active Catalysts

**Figure 2:**
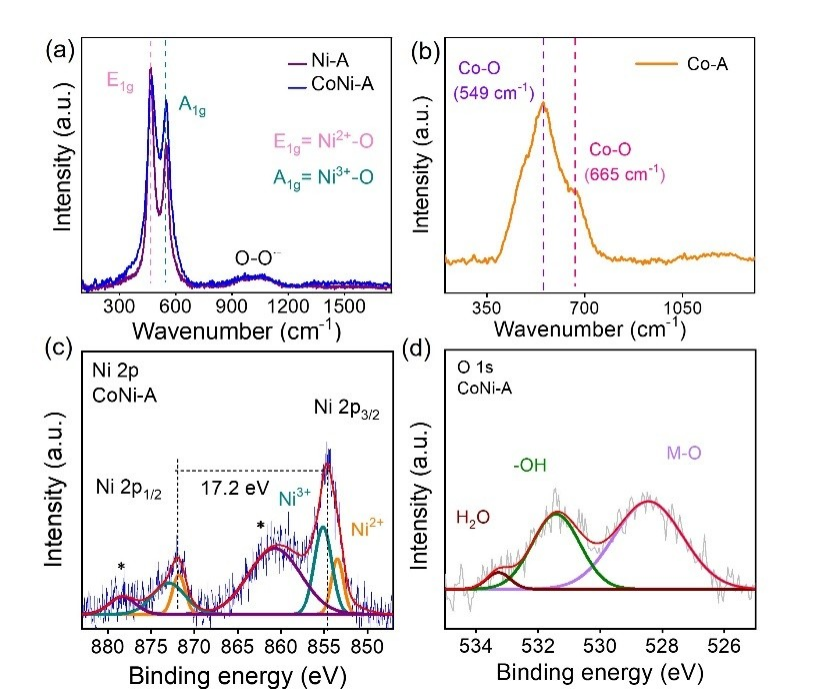
(a) Raman spectrum of CoNi−A and Ni−A showing two intense peaks for Ni^2+^−O (E_1g_) and Ni^3+^−O (A_1g_) vibrations and a broad peak between 850 to 1100 cm^−1^ for superoxide species. (b) Raman spectrum of Co−A displaying two peaks at 549 cm^−1^ and 665 cm^−1^, corresponding to the Co−O bond. (b) Ni 2p XPS of CoNi−A demonstrating peaks for Ni^3+^ and Ni^2+^. (c) O 1s XPS of CoNi−A deconvoluted into peaks at 528.6 eV (Ni−O), 531.3 eV (−OH), and 533.2 eV (adsorbed H_2_O).

The PXRD of CoNi−A and Ni−A revealed an amorphous nature of the catalyst (Figure S5), while the diffraction peaks of Co−A match with β‐Co(O)OH and α‐Co(OH)_2_. The Raman spectra of CoNi−A and Ni−A showed two strong vibrations for Ni^3+^−O and Ni^2+^−Ocorresponding to the A_1g_ stretching vibration and E_g_ bending vibration, respectively (Figure 2a).[^50^, ^51^] In CoNi−A, a positive shift of both A_1g_ (+3 cm^−1^) and E_g_ (+5 cm^−11^) peaks were observed compared to that of Ni−A. The intensity ratio of E_g_ to A_1g_ peak for CoNi−A and Ni−A was calculated to be 1.23 and 1.50, respectively, indicating the atomic‐level thickness of CoNi−A nanosheets.^[52]^ Further, the intensity ratio indicated the presence of higher amount of Ni^3+^–O species in CoNi−A. Interestingly, a broad peak appeared in the high wavenumber region of 850–1150 cm^−1^, attributed to O−O^⋅−^ species present in the structure of (oxy)hydroxides (Figure 2a). For Co−A, two bands were observed at 552 cm^−1^ and 670 cm^−1^, corresponding to Co−O vibrations. (Figure 2b).^[53]^

**Figure 3:**
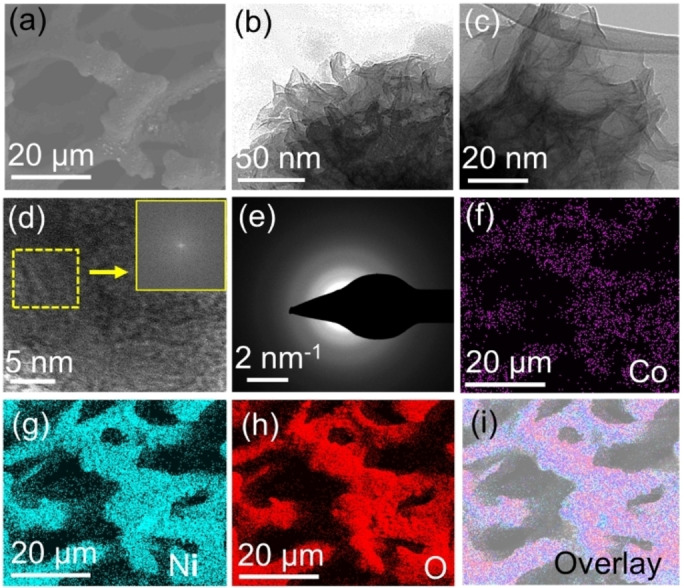
(a) SEM image of CoNi−A on NF; (b‐c) TEM images of CoNi−A at different resolutions; (d) HRTEM image of CoNi−A cannot detect any lattices; inset FFT showing the amorphous nature of CoNi−A; (e) SEAD pattern further confirms the amorphous nature of CoNi−A; (f) elemental mapping of CoNi−A showing (f) Co K‐edge, (g) Ni K‐edge, (h) O K‐edge, and (i) their overlapping image.

The electronic environment around the elements in the active catalysts was examined by X‐ray photoelectron spectroscopy (XPS) (Figure 3). The Ni 2p XPS of CoNi−A and Ni−A was deconvoluted into two peaks for Ni 2p_3/2_ and Ni 2p_1/2_ (Figure 2c).[^46^, ^54^, ^55^] The Ni 2p_3/2_ peak of CoNi−A was further fitted into two peaks for Ni^2+^ (BE: 854.3 eV) and Ni^3+^ (BE: 855.8 eV). The positive shift of 0.5 eV in the binding energy of the Ni 2p_3/2_ peak in CoNi−A suggests a higher positive charge density on Ni compared to that in Ni−A (Figure S6).

The Ni 2p_3/2_–2p_1/2_ spin‐orbit coupling also showed the same conclusion (Figure S6). The peak area ratio (Ni^3+^/Ni^2+^) in CoNi−A (1.9) was found to be significantly higher than that of Ni−A (0.6), clearly indicating different electronic structures of Ni in the two active catalysts.^[56]^ In CoNi−A, the increase in the valence state of Ni compared to Ni−A can be explained by the facile oxidation of Co^2+^ at a lower anodic potential. This enhances the charge transfer, accelerating the faster oxidation of Ni^2+^ to Ni^3+^.

The Co 2p XPS data of CoNi−A is extremely poor because of the leaching of Co during the anodic activation of PBA (Figure S7).[^57^, ^58^, ^59^] The amount of Co in the active catalyst was determined to be only 2.5 % of Ni, which indicates substantial leaching of Co during the anodic activation process. The O 1s spectra of CoNi−A and Ni−A were fitted with three peaks for M−O, ‐OH, and adsorbed H_2_O molecules. (Figure 2d and S8).

The Co 2p XPS of Co−A exhibited two peaks for Co 2p_1/2_ and Co 2p_3/2_. The Co 2p_3/2_ peak was fitted into two peaks, corresponding to Co^2+^ at 783.3 eV and Co^3+^ at 781 eV. (Figure S9).[^60^, ^61^] The Co 2p_3/2_–2p_1/2_ spin‐orbit coupling value was determined to be 15.6 eV, indicating the presence of mixed valent Co^3+^ and Co^2+^ species. Furthermore, the peak area ratio of Co^3+^/Co^2+^ (1.07) showed the presence of a higher amount of Co^3+^ than that of Co^2+^ in Co−A.[^60^, ^61^]

The O1s XPS peak was deconvoluted into three peaks corresponding to M−O (528.9 eV), surface ‐OH (531.2 eV), and adsorbed H_2_O (532.1 eV) species (Figure S10).^[48]^ Therefore, the Raman and XPS data confirmed the presence of Co−Ni(O)OH in CoNi−A with a higher amount of Ni^3+^ species. In contrast, Co−A contains Co(O)OH with the presence of Co^2+/3+^ ions. Furthermore, SEM images showed the uniform distribution of CoNi−A and Co−A on the NF (Figure 3 and S11).^[13]^ The TEM and HR‐TEM images detected the amorphous nature of CoNi−A. In the SEAD pattern, well‐defined diffraction rings were also not visible because of the amorphous nature of CoNi−A (Figure 3).

The elemental mapping of CoNi−A showed a uniform distribution of the elements Co, Ni, and O in the catalyst film (Figure 3). Energy‐dispersive X‐ray (EDX) spectroscopy detected the presence of Co, Ni, and O (Figure S12). After activation, the Co to Ni ratio was decreased substantially, which is consistent with the XPS data (Table S1). Figures S11 and S12 show the EDX spectrum and elemental mapping of Co−A.

### 2.4 Electrochemical OER Activity

**Figure 4:**
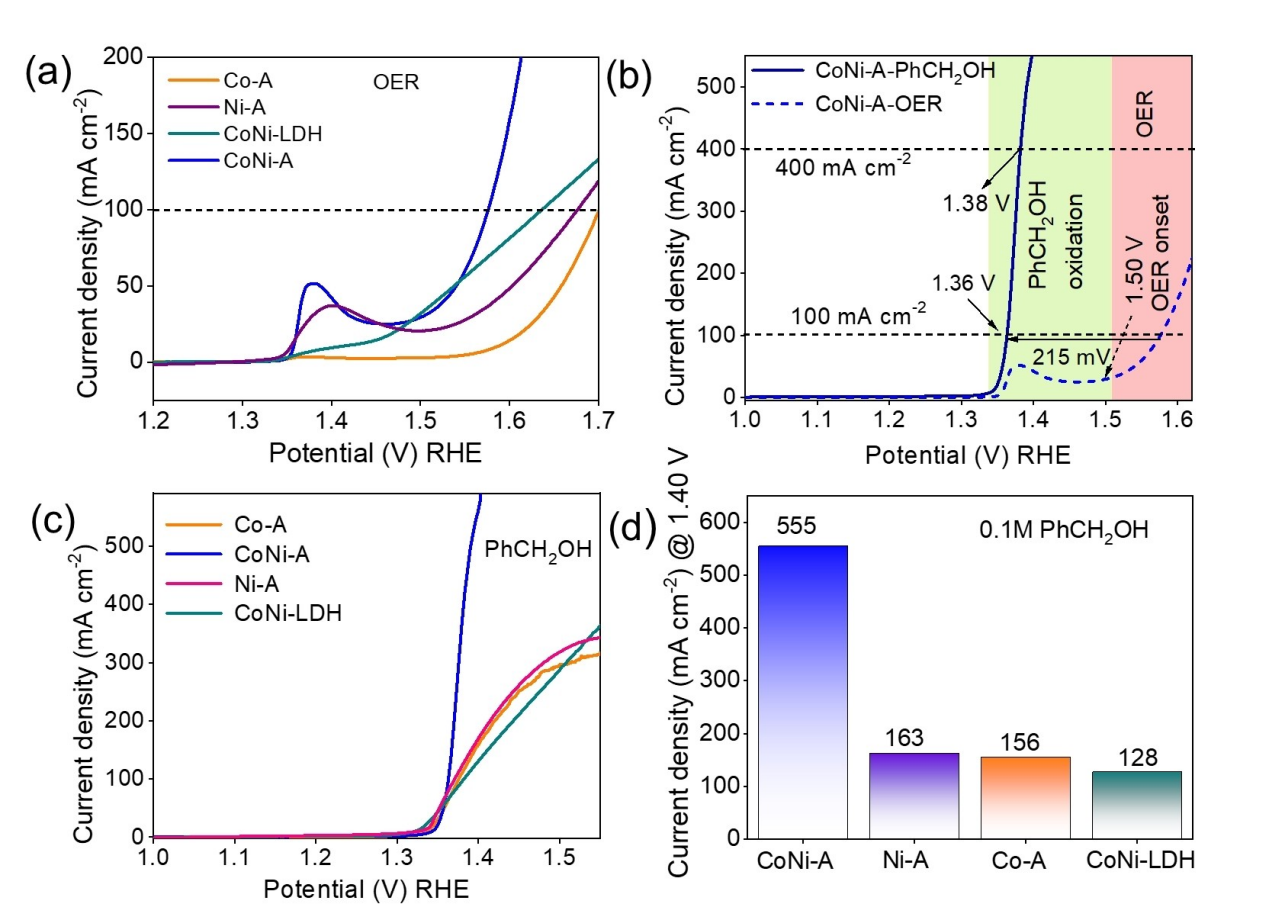
(a) LSV profiles for OER with CoNi−A, Ni−A, Co−A, and CoNi‐LDH (1.0 M KOH solution and a scan rate of 5 mV s^−1^); (b) The LSV profile of CoNi−A demonstrates improved potential requirements and higher current density after the addition of PhCH_2_OH, achieving industrial current density (≥400 mA cm^−2^) at very low electrochemical potential (1.38 V vs. RHE); (c) LSV profiles of CoNi−A, Ni−A, Co−A, and CoNi‐LDH for PhCH_2_OH oxidation (1 M KOH + 0.1 M PhCH_2_OH at a scan rate of 5 mV s^−1^); (d) Comparison of current densities (at 1.40 V) of CoNi−A, Co−A, Ni−A, and CoNi‐LDH in 0.1 M PhCH_2_OH;

The electrochemical performance of CoNi−A, Ni−A, and Co−A was evaluated in a standard three‐electrode system (scan rate 5 mv/s) (Figure 4a). In the CV profile of CoNi−A, a prominent oxidation wave was observed at 1.36 V vs. RHE, corresponding to the Ni^2+^/Ni^3+^ oxidation (Figure S13). Similarly, the Ni−A displayed a Ni^2+/^Ni^3+^ oxidation peak at a slightly higher potential of 1.39 V vs. RHE, indicating the presence of Co as a hetero atom in CoNi−A lowered the potential of electrochemical anodic Ni^2+^ oxidation. The superior electrochemical activity of CoNi−A over Co−A and Ni−A can be explained by the facile oxidation of Ni^2+^ to Ni^3+^. Recent reports show that the oxidation of Ni^2+^ to Ni^3+^ occurs between the potential range of 1.30 to 1.40 V vs RHE, while the oxidation of Co^2+^ to Co^3+^ takes place between 1.10 to 1.30 V vs RHE and Co^3+^ to Co^4+^ at higher than 1.40 V vs. RHE.^[41]^ In CoNi−A, the oxidation of Co^2+^ to Co^3+^ was not observed due to its low concentration. In contrast, the oxidation peaks in the case of Co−A were clearly visible between 1.37 to 1.42 V vs. RHE due to the oxidation of Co^2+^ to Co^3+/4+^ (Figure S13).

Furthermore, the LSV profile showed CoNi−A achieved a current density of 100 mA cm^−2^ at an overpotential of 340 mV, whereas Co−A, Ni−A, and CoNi‐LDH (hydrothermally prepared) reached the same current density at overpotentials of 460 mV, 430 mV and 460 mV, respectively (Figure 4a). These results show that the electrochemically accessed CoNi−A produces superior OER activity.

Tafel slopes of active the catalysts CoNi−A, Ni−A, and Co−A were calculated to be 102 mV dec^−1^, 115 mV dec^−1,^ and 128 mV dec^−1^, respectively, revealing a faster OER kinetics with CoNi−A (Figure S14).^[59]^ Furthermore, electrochemical impedance spectroscopy (EIS) revealed a lower charge‐transfer resistance for CoNi−A than that of Co−A (Figure S15).^[62]^ The electrochemical surface area (ECSA) was determined by measuring double‐layer capacitance (*C*_dl_, which is proportional to ECSA) through CV cycles in the non‐Faradaic region at various scan rates. The CoNi−A showed slightly higher *C*_dl_ (4.1 mF cm^−2^) than Co−A (3.3 mF cm^−2^) and Ni−A (3.7 mF cm^−2^) (Figure S16).[^62^, ^63^, ^64^] Similarly, CoNi−A showed the highest number of active sites among the PBA derived active catalysts (Figure S17). The active sites normalized activity showed the highest value for CoNi−A (Figure S18).^[64]^ Additionally, a long‐term chronoamperometry test was conducted for CoNi−A in 1.0 M KOH to evaluate its stability. CoNi−A exhibited no significant decay of current for 22 hours (Figure S19).

### 2.5 Electrocatalytic PhCH_2_OH Oxidation

To find a possible way to replace the OER with more facile processes, we performed electrochemical oxidation of PhCH_2_OH using Co−A, Ni−A, and CoNi−A (Electrolyte;1 M KOH + 0.1 M PhCH_2_OH, scan rate; 5 mV/s).

CoNi−A exhibited a current density of 100 mA cm^−2^ at 1.36 V vs. RHE, which is 215 mV lower than the potential required for the OER at the same current density (Figure 4b). Furthermore, the addition of PhCH_2_OH reduces the anodic onset potential, indicating a significant improvement in energy efficiency. In contrast, the other catalysts, Co−A, Ni−A, and hydrothermally prepared CoNi‐LDH showed lower current densities at the same potential (Figure 4c and d). At lower potentials (<1.5 V vs RHE), Co−A reached higher current densities than CoNi‐LDH. However, at the potential >1.5 V vs. RHE, the current density of CoNi‐LDH increased rapidly and surpassed that of Co−A.

Among the catalysts, only CoNi−A and CoNi‐LDH achieved industrial‐scale current density (≥ 400 mA cm^−2^). CoNi‐LDH reached this current density at 1.57 V vs. RHE, while CoNi−A achieved it at 1.38 V vs. RHE, confirming the superior AOR activity of CoNi−A compared to hydrothermally prepared CoNi‐LDH. This also indicates that the amount of Ni is not the primary factor behind the higher electrochemical activity of CoNi−A in OER and PhCH_2_OH oxidation. If the amount of Ni were the only factor, the best catalytic activity should be achieved with Ni−A. The product yield with Ni−A was only 65 % with a Faradaic efficiency of 98 % under similar reaction conditions. The poor yield with Ni−A further confirmed the importance of structural modulation of Ni(O)OH for PhCH_2_OH oxidation.

For CoNi−A, the OER onset potential was 1.50 V vs. RHE, but after the addition of PhCH_2_OH, the onset potential shifted positively by 215 mV. The industrial current density CoNi−A is also achieved at a lower potential than the onset potential of OER, indicating no interference from OER.

In alcohol‐free electrolyte, a high‐intensity oxidation peak was observed at 1.36 V vs. RHE, attributed to the oxidation of Ni^2+^ to Ni^3+^. However, after the addition of PhCH₂OH, this oxidation peak was no longer observed, suggesting the reduction of Ni^3+^ to Ni^2+^ by PhCH₂OH. The chronoamperometric (CA) oxidation of PhCH_2_OH was recorded at 1.39 V vs. RHE (Figure S20). Only 3 hours were required for a complete conversion of 0.1 M PhCH_2_OH to PhCOOH. The product yield and FE for PhCOOH formation were found to be 95 % and 97 %, respectively (Figure S20). The stability of the catalyst was evaluated by conducting five consecutive CA batches. Even after five CA batches, the catalyst maintained a high FE (≥95 %) and PhCOOH yield, indicating its robust stability (Figure S21).

After PhCH_2_OH oxidation, we characterized the catalyst CoNi−A. The PXRD detected that the amorphous structure of CoNi−A remained unchanged after PhCH_2_OH oxidation (Figure S22). The CA conversion of PhCH_2_OH to PhCOOH was evaluated for the other three catalysts, Co−A, Ni−A, and CoNi‐LDH, and the PHCOOH yields were observed to be <70 % after the same reaction time (3 h) (Figure S20).

After the complete passing of charge 951 C, the oxidized product was isolated by neutralization and hot water crystallization. The ^1^H and ^13^C NMR (Figures S23 and S24) confirmed the formation of benzoic acid as the single product.

Additionally, we compared the activity of our synthesized catalyst with that of recently reported catalysts (Table S2). We observed that the potential at which PhCH_2_OH oxidation occurred ranged between 1.40 V vs RHE to 1.5 V vs RHE, with only a few demonstrating good activities. Interestingly, previously we have also reported FeCo(O)OH for the electrochemical oxidation of various alpha‐hydrogen‐containing benzyl derivatives, with a primary focus on PhCH_2_OH derivatives. We observed that the oxidation was completed in 3 hours at a potential 1.50 V vs RHE.^[37]^ In this paper, the oxidation of PhCH_2_OH was achieved at potentials below 1.40 V, resulting in a 95 % yield and a 97 % FE. This performance is significantly better than most of the previously reported catalysts, making our catalyst uniquely efficient compared to the others (Table S2).

Furthermore, the performance of CoNi−A, CoNi‐LDH, and Co−A was evaluated for the anodic oxidation of two aliphatic alcohols (ethylene glycol and methanol). The LSV profiles showed that CoNi−A achieved a higher current density compared to Co−A and CoNi‐LDH for both aliphatic alcohols (Figure 4c and S25). This suggests that CoNi−A exhibits superior electrochemical activity even with aliphatic alcohols. Additionally, when comparing the onset potential, CoNi−A demonstrated a lower electrochemical potential than the other catalysts (Figure 4c and S26). Moreover, the comparison of the potential requirement for the oxidation of the three alcohols with CoNi−A revealed the most favorable oxidation potential for PhCH_2_OH.

Previous studies on Ni‐based electrocatalysts for alcohol and aldehyde oxidation have revealed that the commonly accepted mechanism involves the in‐situ formation of redox‐active Ni^3+^(O)OH intermediates.^[65]^ Based on these findings, further the reaction mechanism was evaluated.^[65]^ In the CV curves, the oxidation of Ni^2+^(OH)_2_ to Ni^3+^(O)OH was observed at 1.36 V vs RHE in KOH solution. When PhCH_2_OH was added, this peak was disappeared, confirming that Ni(O)OH was involved in the PhCH_2_OH oxidation process.

Initially, Co^2+^/Ni^2+^ species are oxidized to Co^3+^/Ni^3+^ by the applied anodic potential. The OH^−^ ions then deprotonate the M^3+^‐μ‐OH‐M^3+^ intermediate, initiating the oxidation process.^[37]^ Subsequently, gradual oxidation and deprotonation produce the Ph‐COH* intermediate. In the final step, Ph‐COH* undergoes further attack by OH^−^ to form PhCOOH. Throughout the process, μ‐OH plays a crucial role in facilitating electron transfer, deprotonation, and substrate binding.^[37]^ The metal centres mediate electron transfer from the electrode, driving the overall reaction.

The higher electrochemical activity for Ni‐containing catalyst can be by the facile oxidation of Ni^2+^ to Ni^3+^, which facilitates the oxidation of alcohols.[^66^, ^67^] The faster electrooxidation kinetics with Ni‐based catalysts can be addressed by the facile nucleophilic attack and electron transfer through the vacant orbitals of trivalent nickel (t_2g_^6^ e_g_^1^).[^68^, ^69^]

## 3 Conclusions

In summary, we have developed Co‐doped Ni(O)OH (CoNi−A) nanosheets by the electrochemical activation of PBA. The electrochemically accessed CoNi−A exhibits excellent electrocatalytic activity for PhCH_2_OH oxidation, with ≥95 % product yields and FE. The catalyst CoNi−A reaches industrial‐level current density at a potential of 1.38 V vs. RHE, which is superior to most of the reported catalysts. Most importantly, the superior activity of NiCo−A is not only limited to PhCH_2_OH oxidation but it can be extended for the anodic oxidation of the aliphatic alcohols like methanol and ethylene glycol with a high FE. The superior electrochemical activity of CoNi−A compared to Ni−A can be attributed to the facile oxidation of Ni^2+^ to Ni^3+^.

## Conflict of Interests

The authors declare no conflict of interest.

4

## Supporting information

As a service to our authors and readers, this journal provides supporting information supplied by the authors. Such materials are peer reviewed and may be re‐organized for online delivery, but are not copy‐edited or typeset. Technical support issues arising from supporting information (other than missing files) should be addressed to the authors.

Supporting Information

## Data Availability

The data that support the findings of this study are available from the corresponding author upon reasonable request.
