# Supplementary material for: Anodic Activation of Prussian Blue Analog Leads to Highly Active Cobalt‐Doped Nickel (Oxy)Hydroxide for Organic Oxidation Reactions
Source: Chemistry. 2025 Jan 13;31(14):e202404174. doi: 10.1002/chem.202404174 (PMC11886769; doi:10.1002/chem.202404174)
Supplement: Supplementary file 1 — Supporting Information [file CHEM-31-e202404174-s001.pdf]

Chemistry–A European Journal

Supporting Information

Anodic Activation of Prussian Blue Analog Leads to Highly Active Cobalt-Doped Nickel (Oxy)Hydroxide for Organic Oxidation Reactions

Toufik Ansari, Debabrata Bagchi, Suptish Ghosh, Jan Niklas Hausmann, Arindam Indra,* and Prashanth W. Menezes*

Supporting Information

Anodic Activation of Prussian Blue Analog Leads to Highly Active Cobalt-doped Nickel (Oxy)hydroxide for Organic Oxidation Reactions

Toufik Ansari,[a] Debabrata Bagchi,[b] Suptish Ghosh,[c] Jan Niklas Hausmann,[b] Arindam Indra*[a], Prashanth. W. Menezes*[b,c]

[a] Toufik Ansari and Prof. Arindam Indra,

Department of Chemistry, IIT (BHU), Varanasi, UP-221005, India

E-Mail: arindam.chy@iitbhu.ac.in

[b] Dr. Debabrata Bagchi, Dr. Jan Niklas Hausmann and Dr. Prashanth W. Menezes

Material Chemistry Group for Thin Film Catalysis

CatLab, Helmholtz-Zentrum Berlin für Materialien und Energie

Albert-Einstein-Str. 15, 12489 Berlin, Germany

E-Mail: prashanth.menezes@helmholtz-berlin.de

[c] Suptish Ghosh and Dr. Prashanth W. Menezes

Department of Chemistry, Metalorganics and Inorganic Materials,

Technische Universität Berlin, Straße des 17 Juni 115, Sekr. C2, 10623 Berlin, Germany

E-Mail: prashanth.menezes@mailbox.tu-berlin.de

Chemicals

The reagents were obtained and used as received without further purification. $Ni(NO_3)_2.6H_2O$ (≥ 98.5%), $Co(NO_3)_2.6H_2O$ were procured from SRL India while potassium hexacyanocobaltate (III) (> 99.0 %) and potassium tetracyanonickelate(II) were bought from Sigma-Aldrich. Benzyl alcohol, ethylene glycol, and methanol were procured from Merck. Potassium hydroxide was obtained from Sigma. Washing, synthesis, and electrochemical tests were performed by using double-distilled water. Nickel foam was brought from Axys Pvt. limited.

Instruments

The PXRD patterns of the precatalysts and active catalysts were measured in the 2θ range of 5°-80° using a Rigaku D/MAX RINT-2000 X-Ray diffractometer, with Cu-K_{α} radiation (λ = 1.5418 Å).

X-ray photoelectron spectroscopy (XPS) was carried out using VG/VG ESCA LAB 220i X-ray photoelectron spectrometer to determine the chemical nature, oxidation state, and surface structure of the synthesized catalysts. SEM images were recorded using two different systems. The first system employed a Thermo Scientific Quattro S SEM with an accelerating voltage of 10 kV. The second system, used for mapping and elemental analysis, involved a ZEISS GeminiSEM500 NanoVP microscope equipped with a Bruker Quantax XFlash® 6|60 energy dispersive X-ray (EDX) detector.

Transmission electron microscopy (TEM) was conducted using an FEI Tecnai G2 20 S-TWIN microscope (FEI Company, Eindhoven, Netherlands) equipped with a LaB6 source and operated at an acceleration voltage of 200 kV.

A Thermo Scientific Nicolet iS5 FTIR spectrometer was used to record IR spectra of the synthesized catalysts.

Activation of nickel foam (NF)

First, the foam was cut into 1x2 cm^2 pieces and sonicated in a 1.0 M HCl solution for 15 minutes. Then, the pieces were sonicated in distilled water for an additional 15 minutes. After sonication, the nickel foam pieces were washed with acetone and dried in an air oven at 60 °C for 3 hours.

Synthesis

Preparation of NiCo-PBA

For the synthesis of NiCo-PBA, 1.5 mmol of nickel nitrate hexahydrate was dissolved in 25 mL of deionized water to form solution A. In a separate beaker, 1 mmol of potassium hexacyanocobaltate (III) was dissolved in 25 mL of deionized water to form solution B. Solution B was then added dropwise to solution A under magnetic stirring for 5 minutes. The resulting mixture was allowed to age at room temperature for 24 hours. The blue product was collected via centrifugation, washed multiple times with deionized water, and dried in a hot air oven at 60 °C overnight.

Preparation of CoCo-PBA

1 mmol potassium hexacyanocobaltate(III) solution in 25 mL water was added to the solution of cobalt nitrate hexahydrate (1.5 mmol in 25 mL water) and stirred for 5 minutes. The resulting mixture was kept at room temperature for 24 hours without any stirring. The pink precipitate was collected via centrifugation, washed with deionized water, and dried in a hot air oven at 60 °C overnight.

Preparation of NiNi-CP

1 mmol potassium tetracyanonickelate(II) solution in 25 mL water was added to the solution of nickel nitrate hexahydrate (1 mmol in 25 mL water) and stirred for 5 minutes. The resulting mixture was kept at room temperature for 24 hours without any stirring. The precipitate was collected via centrifugation, washed with deionized water, and dried in a hot air oven at 60 °C overnight

Electrophoretic deposition (EPD) of the precatalysts on NF

The catalysts were electrophoretically deposited on activated NF. In a two-electrode setup using NF (2×1 cm^2) as the counter and reference electrode and a mixture of iodine and acetone as the electrolyte. In the standard procedure, 25 mg of the respective material was suspended in 15 mL acetone and sonicated at room temperature for 30 minutes. Then, 5 mg of iodine was added, and the suspension was sonicated for three minutes. Immediately after the sonication, EPD was performed at 9 V for 300 s with stirring to obtain uniform films.

Electrochemical transformation of precatalyst into active catalyst

The cyclic voltammetry (CV) was used for the activation of the precatalysts. The activation of the precatalyst was performed in a single-compartment electrocatalytic cell using precatalyst@NF as the working electrode, Hg/HgO as the reference electrode, and Pt wire as the counter electrode in 1.0 M KOH solution. The scan rate used for the CV was 5 mV s^{-1} for the activation process. The names of the active catalysts corresponding to the precatalysts have been mentioned in Table 1.

Electrochemical measurements

Electrocatalytic measurements were performed using a single-compartment three-electrode electrochemical cell containing 20 mL of 1.0 M aqueous KOH solution (pH 13.8). A potentiostat (SP-200, BioLogic Science Instruments), controlled by the EC-Lab v10.20 software package, was used for the measurements. The working electrode: catalyst-loaded NF, a platinum wire counter electrode, and a Hg/HgO reference electrode. All potentials were reported against the reversible hydrogen electrode (RHE) using the following formula:

$E_{RHE} = E_{Hg/HgO} + 0.098 + 0.059pH$

Electrochemical impedance spectroscopic (EIS) measurements were recorded in the frequency range from 0.001 to 100,000 Hz and amplitude of 10 mV. The chronoamperometric measurements (CA) were carried out in 1.0 M aqueous KOH at a constant potential and represented without *i*R compensation.

The Tafel slope was calculated by using the formula:

$\eta = b \log j + a$

Where, η denotes the overpotential (mV), j is the current density (mA cm^{-2}), and b is the Tafel slope (mV dec^{-1}).

Analysis of product formation by NMR

After the complete passing of charge, the oxidized product was isolated by neutralization and hot water crystallization. The organic product formation after the electro-oxidation reaction of $PhCH_2OH$ was analyzed by ^1H and ^{13}C NMR spectroscopy in a 500 MHz JEOLBRUKER BioSpin NMR instrument. The NMR sample was prepared by taking a 15 mg of the reaction product and 550 μL $CDCl_3$ solvent. Both (^{13}C and ^1H) spectra were analyzed using MestReNova software.

Calculation of Faradic efficiency

The Faradaic efficiency was calculated based on the weight of PhCOOH produced and the total charge passed during the electrocatalytic reaction. Since the full conversion of $PhCH_2OH$ to PhCOOH was confirmed by ^1H NMR spectra, the chemical conversion, and Faradic efficiency were calculated using the following equations:

Chemical conversion (%) = [n(product) / n (internal standard)] x 100 (1)

FE (%) = [n (product formed). ne. F / (Q)] x 100 (2)

where **F** is the Faraday constant (96485 C), n is the number of moles of benzoic acid, **ne** is the number of electrons required for the oxidation process and **Q** is the charge (in coulombs) passed through the solution.

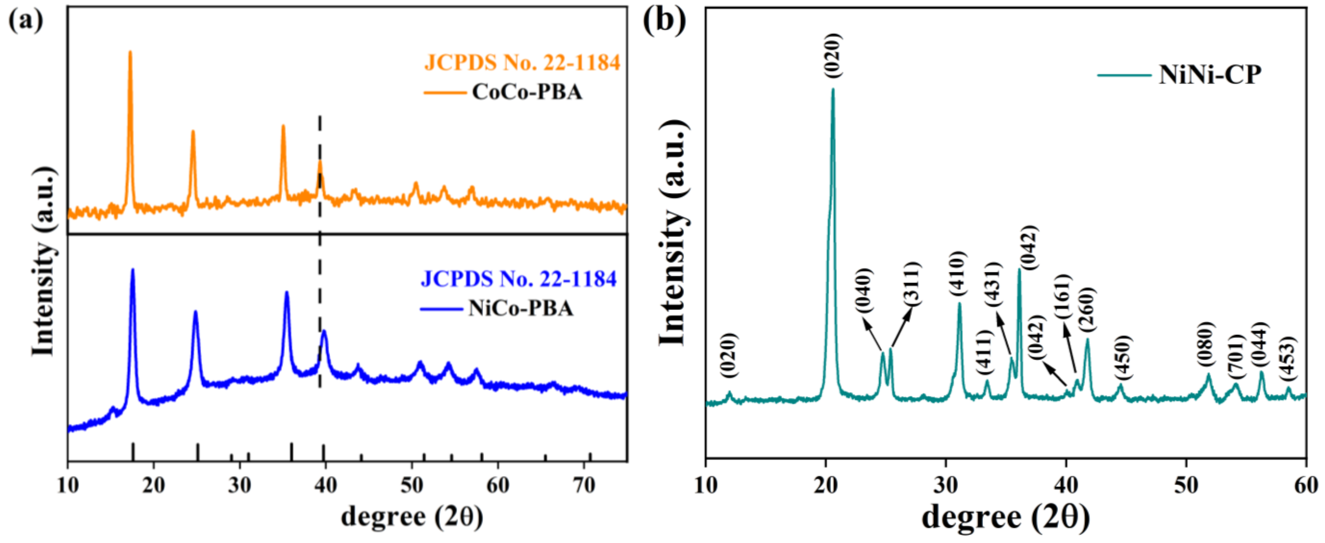

Figure S1. PXRD patterns of (a) NiCo-PBA and CoCo-PBA and (b) NiNi-CP. The peaks of NiCo-PBA and CoCo-PBA were well-indexed and assigned to the cubic crystal system (space group F-43m). The peaks of NiCo-PBA showed a right shift of 0.3-0.4° in the 2-theta value compared to CoCo-PBA. All the peaks are matching with JCPDF No. 22-1184.[1–4] In NiNi-CP, all the diffraction peaks were matched with Hofmann-type $Ni(H_2O)_2[Ni(CN)_4]\cdot xH_2O$.[5]

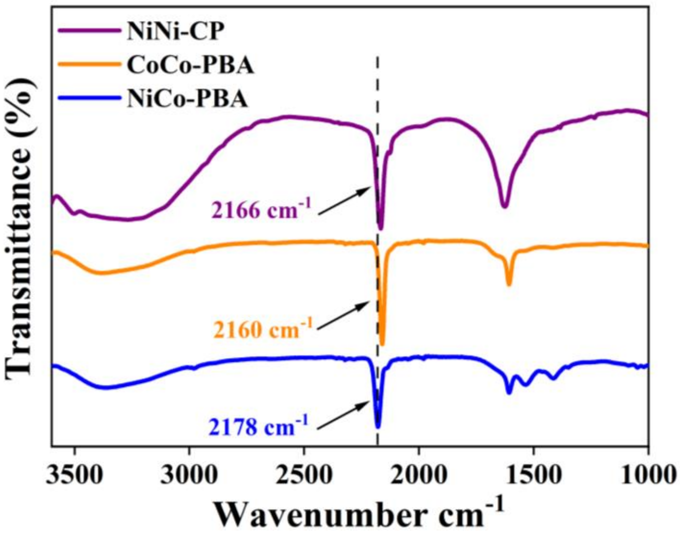

Figure S2. IR spectra of NiNi-CP, CoCo-PBA, and NiCo-PBA.

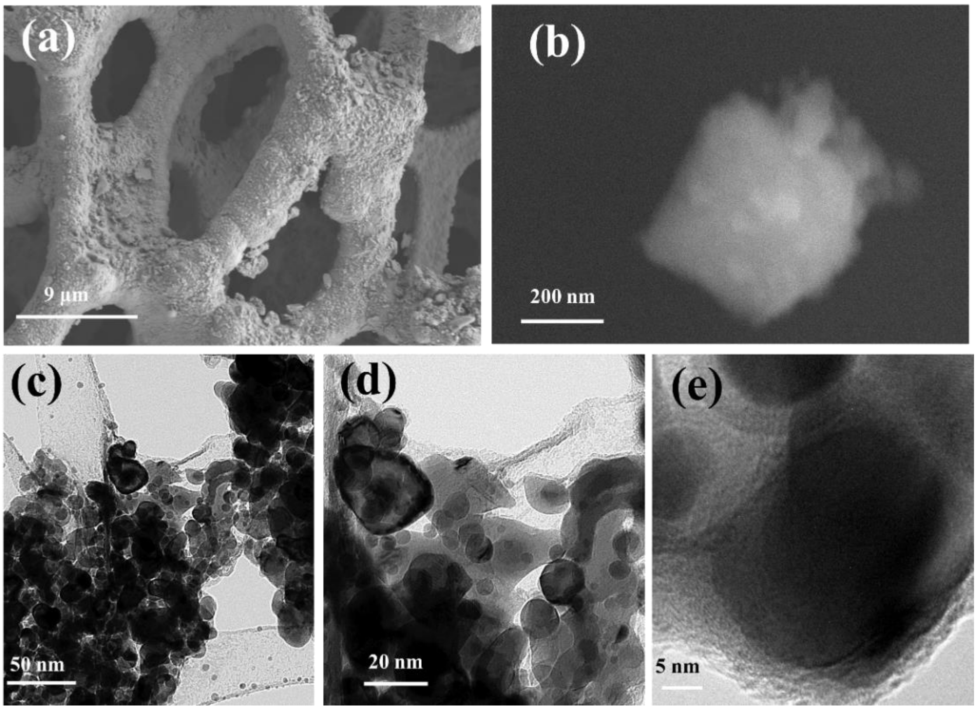

Figure S3. (a) SEM images of NiCo-PBA@NF, (b) nanocube like morphology of NiCo-PBA; (c-e) TEM images with different resolutions.

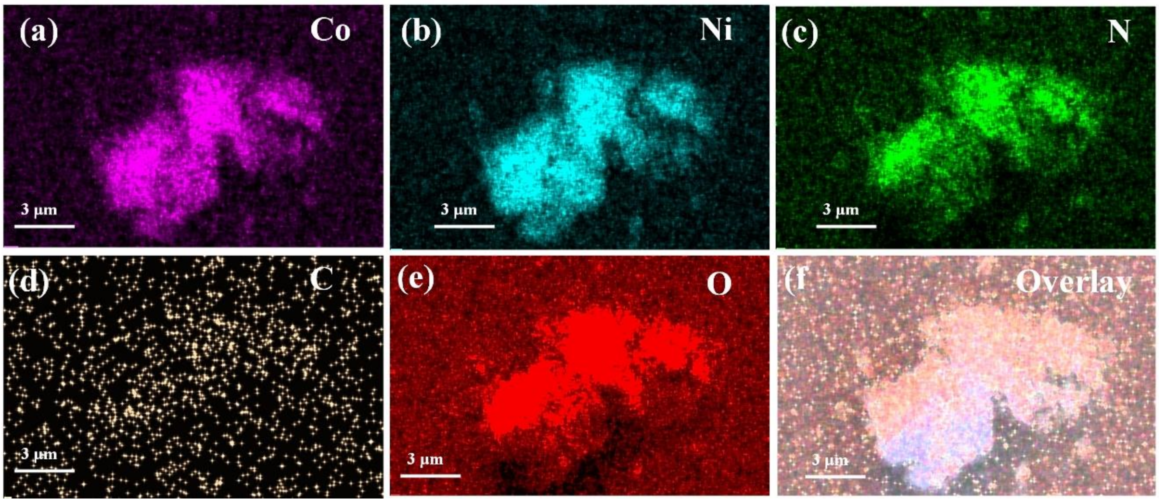

Figure S4. Elemental mapping of powder NiCo-PBA showing Co, Ni, N, C, and O.

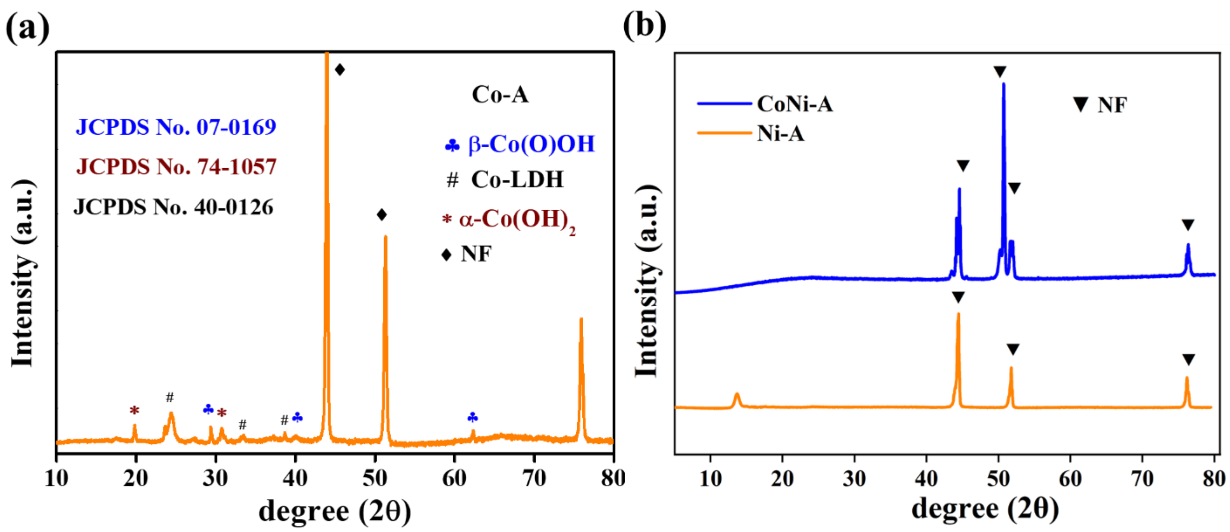

Figure S5. PXRD patterns of the active catalyst Co-A, Ni-A, and CoNi-A. (a) For Co-A, all the peaks are matched with β-Co(O)OH (JCPDF No. 07-0169), Co-LDH (JCPDS No. 40-0126), and α-$Co(OH)_2$ (JCPDF No 74-1057).[9–11] (b) The XRD pattern of CoNi-A reveals no distinct peaks aside from those of the nickel foam, while Ni-A shows a single peak corresponding to the (003) plane of α-$Ni(OH)_2$, indicating the predominantly amorphous nature of the active catalyst.

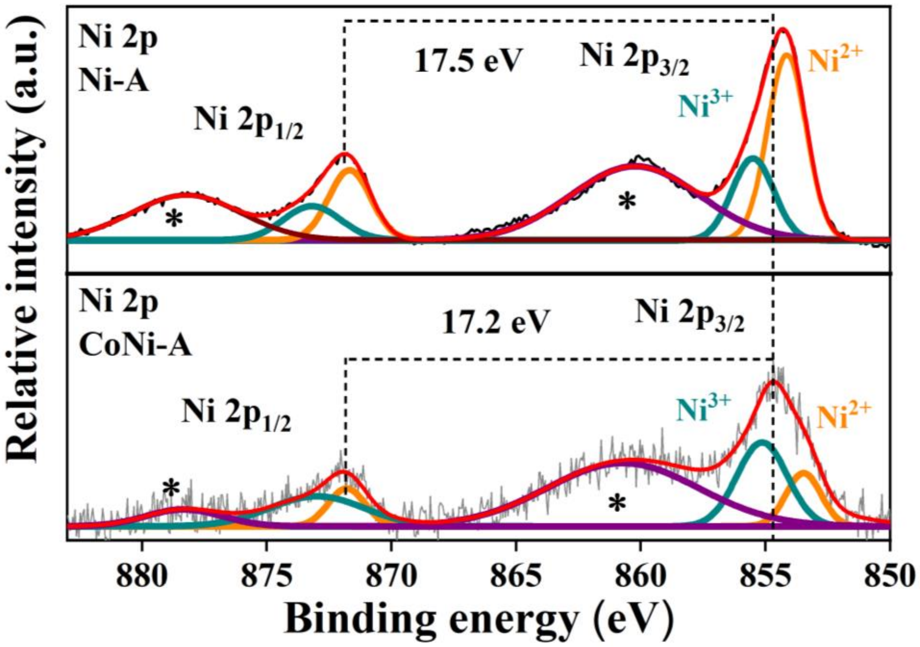

Figure S6. Ni 2p XPS of Ni-A and CoNi-A. The Ni 2p peak of CoNi-A was deconvoluted into two peaks: Ni $2p_{3/2}$ (854.7 eV) and Ni $2p_{1/2}$ (872.9 eV). In contrast, the Ni 2p peak in Ni-A was observed at 854.2 eV and 871. eV corresponding to Ni $2p_{3/2}$ and Ni $2p_{1/2}$ respectively. The positive shift (0.5 eV) of Ni $2p_{3/2}$ peak in CoNi-A indicates a different electronic state in the active catalyst. Additionally, the spin-orbit coupling between the Ni $2p_{3/2}$ and Ni $2p_{1/2}$ peak was calculated to be 17.2 eV for CoNi-A and 17.5 eV for Ni-A.* marked peaks indicate the satellite peaks of Ni^{2+}.[12]

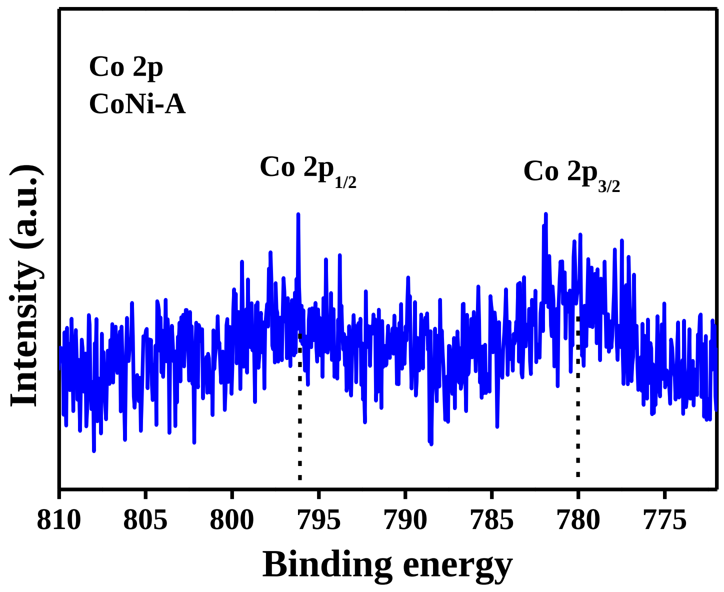

Figure S7. (a) Co 2p XPS of CoNi-A demonstrating the peak of Co $2p_{1/2}$ and Co $2p_{3/2}$.

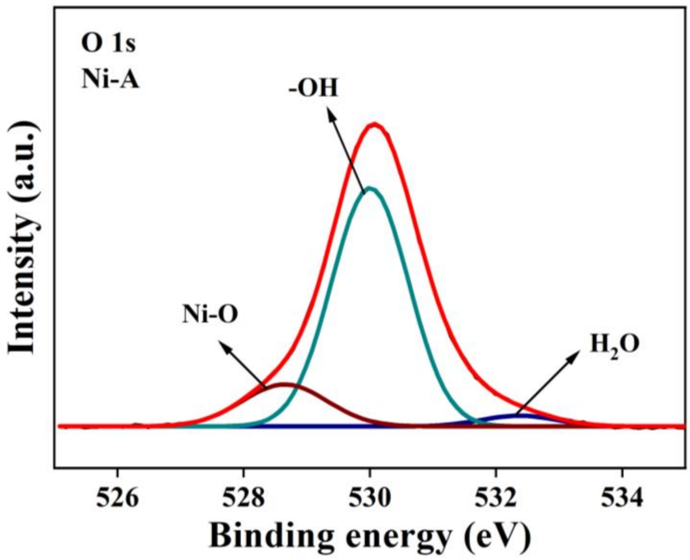

Figure S8. The O 1s XP spectrum of Ni-A was fitted into three peaks. The peaks at 528.6 eV and 530.0 eV were attributed to the Ni-O bond, –OH bond while the peak at 532.4 eV was assigned to adsorbed H_2O.[7,13]

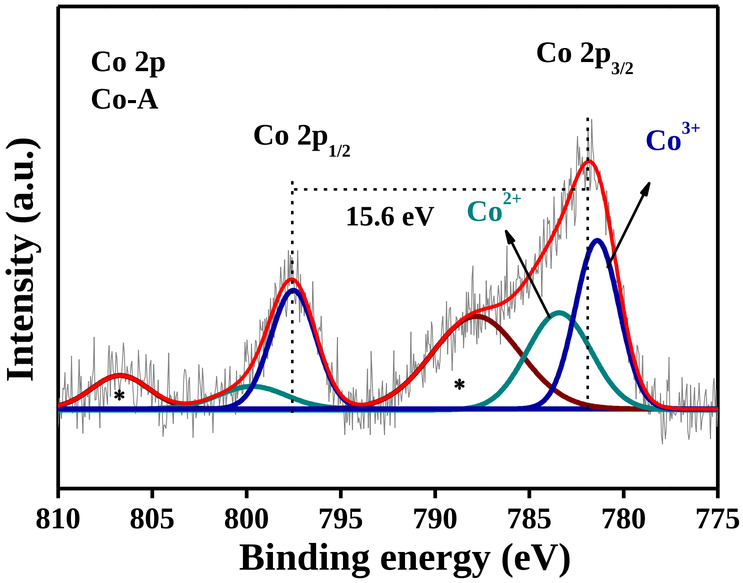

Figure S9. (a) Co2p XPS of Co-A. The spectrum was fitted into two peaks Co $2p_{1/2}$ (790.1 eV) and Co $2p_{3/2}$ (783.1 eV). The peak mentioned at 781.3 eV demonstrating the peak of Co^{3+} while the peak cantered at 783.6 eV is attributed to Co^{2+}. * Indicates the satellite peak.[4,9]

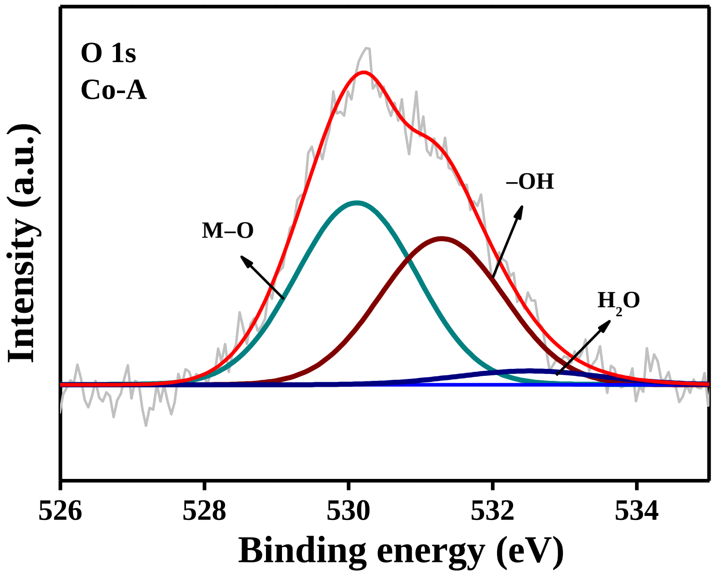

Figure S10. The O 1s XP spectrum of Co-A was fitted into three peaks. The peaks at 530.1 eV and 531.3 eV were attributed to the Co-O bond, –OH bond while the peak at 533.2 eV was assigned to adsorbed H_2O [7,13]

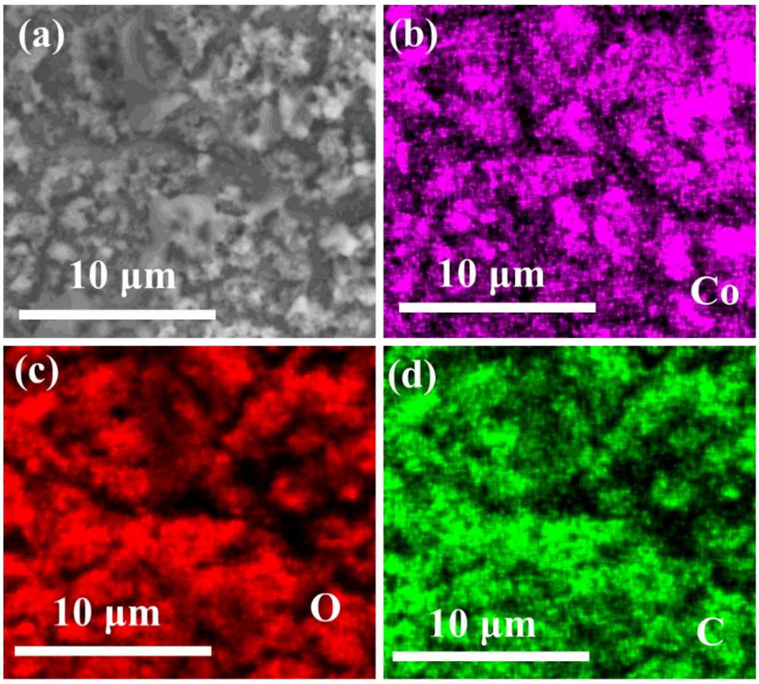

Figure S11. (a) SEM image and (b-d) elemental mapping of Co-A.

(a)

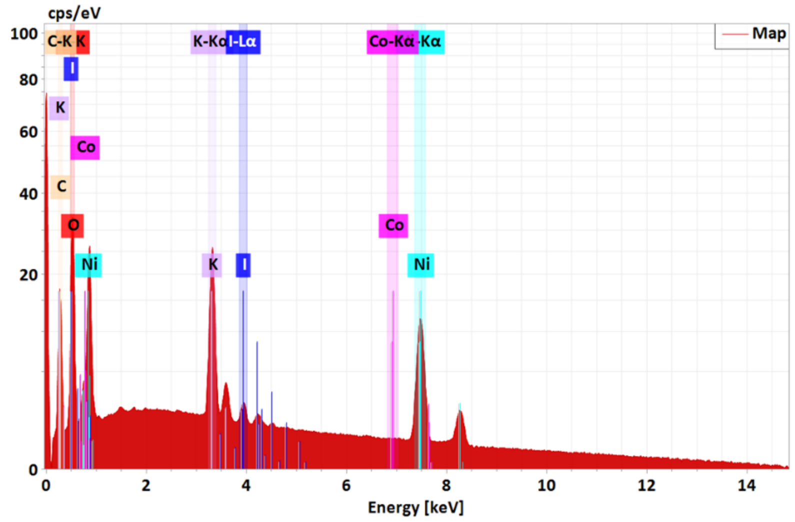

(b)

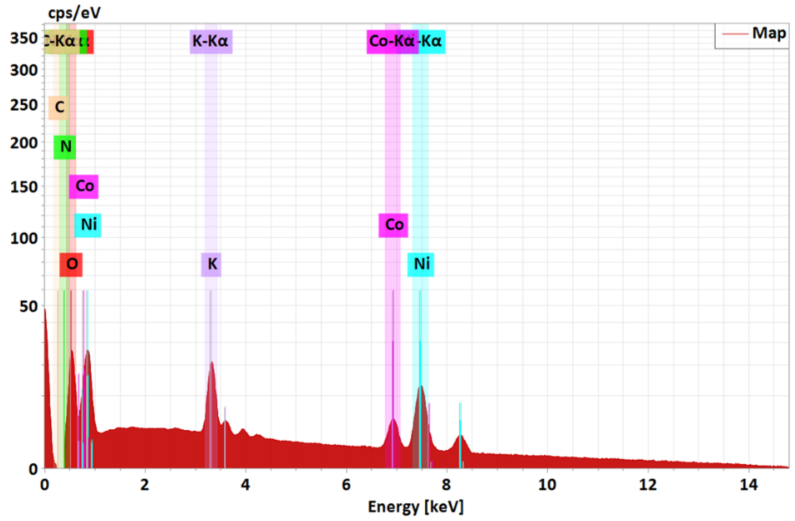

Figure S12. (a) EDX spectrum of CoNi-A displays the presence of Ni, O, and Co. (b) EDX spectrum of Co-A displays the presence of O and Co. The source of potassium (K) is potassium hydroxide (KOH), which is used as an electrolyte, and the residual iodine (I_2) is from electrochemical potential deposition (EPD).

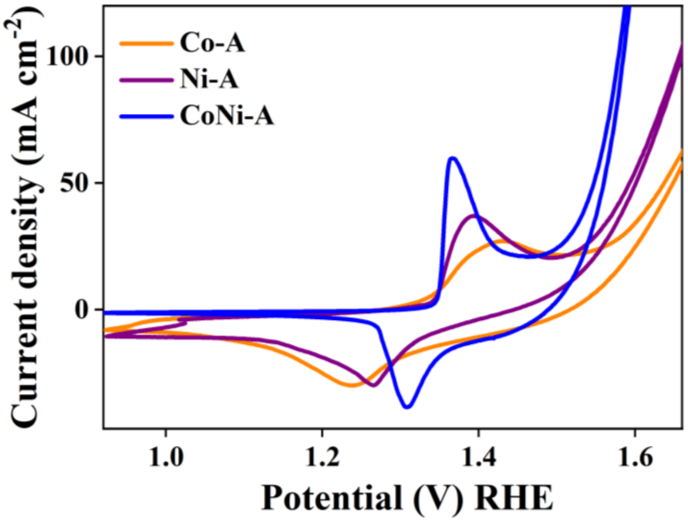

Figure S13. The CV profiles of Co-A, Ni-A, and CoNi-A.

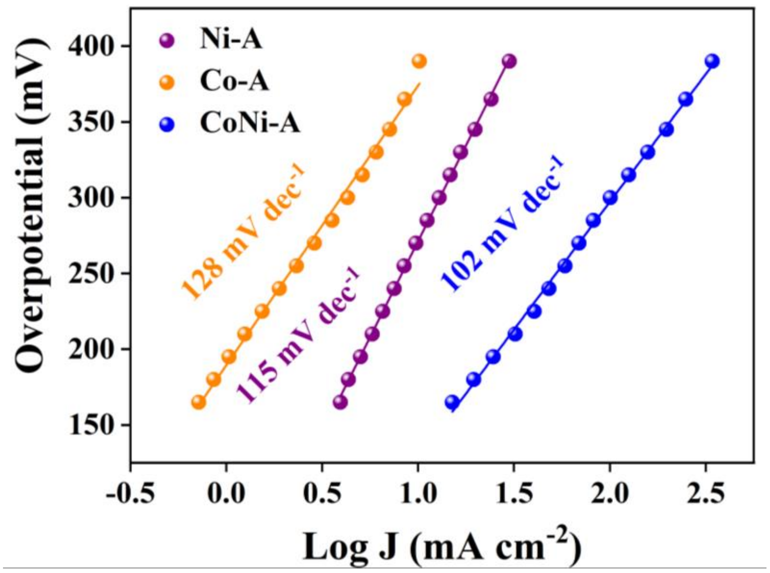

Figure S14. Tafel plots for Co-A, Ni-A, and CoNi-A. A lower Tafel value for CoNi-A indicates faster kinetics.

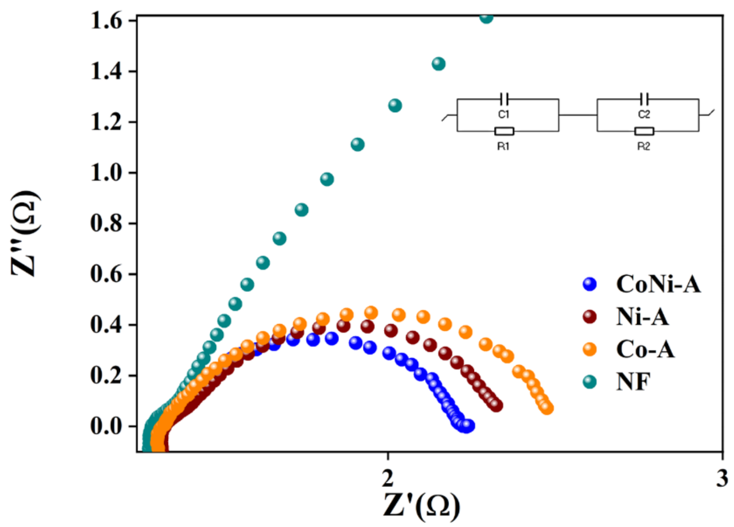

Figure S15. EIS plots of CoNi-A, Ni-A, and Co-A showing the lower R_{ct} for CoNi-A.

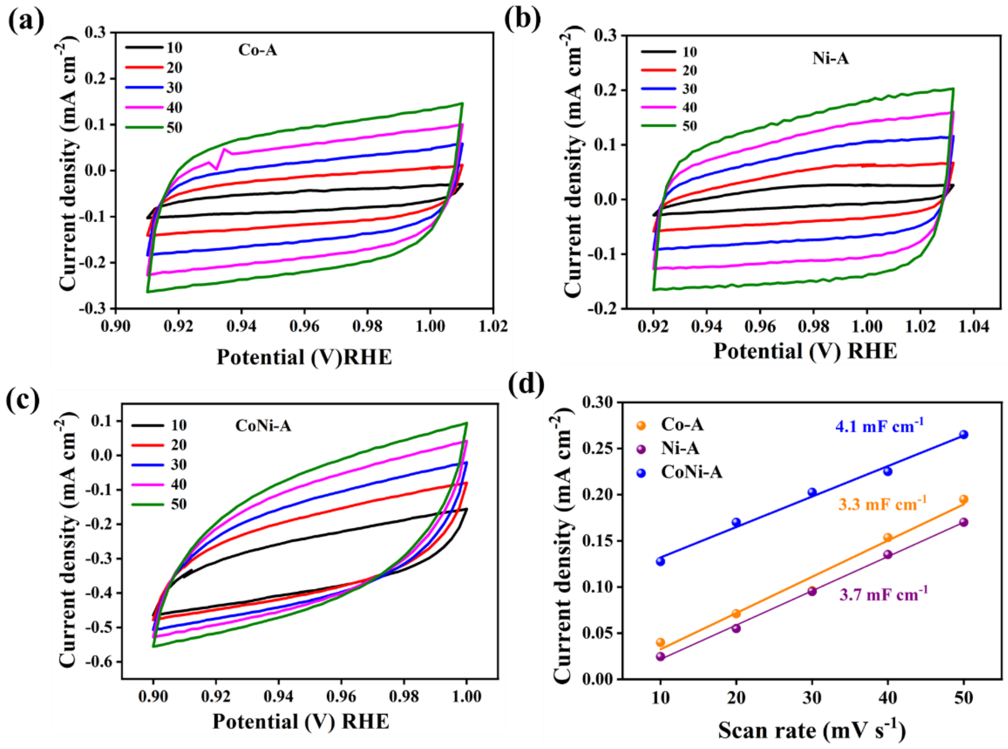

Figure S16. (a-c) CV plots for Co-A, Ni-A, and CoNi-A in the non-faradaic region; (c) electrochemical double-layer capacitance C_{dl} value for CoNi-A, Ni-A, and Co-A.

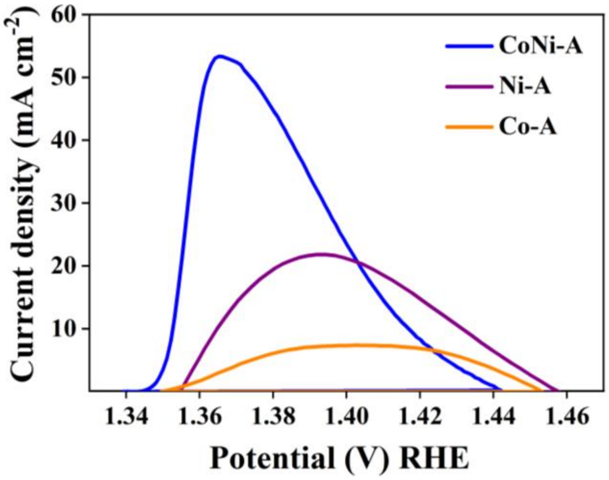

Figure S17: The oxidation peak area of the catalysts CoNi-A, Ni-A, and Co-A, used to determine the number of active sites.

Determination of surface-active sites using area integration of oxidation peak

The ECSA of a catalyst sample is calculated from the double-layer capacitance according to

ECSA = C_{dl}/C_s

C_s = 0.04 mF cm^{-2} in 1 M KOH based on typical reported values.

Where C_{dl} is the double layer capacitance of the catalyst and Cs is the specific capacitance of the material per unit area under identical electrolyte conditions.

CoNi-A

Calculated area associated with the reduction peak = 2.28 x 10^{-6} V A

Hence the associated charge is = 2.28 x 10^{-3} V A / 0.005 V s^{-1}

= 456 x 10^{-3} A s

= 456 x 10^{-3} C

Now, the number of electron transferred is = 456 x 10^{-3} C / 1.602 ×10^{-19} C

= 284.64 x 10^{16}

Since the reduction of Ni^{2+} to Ni^{3+} is a single electron transfer reaction, the number of electrons calculated above is the same as the number of surface-active sites.

Hence,

The surface-active site that participated in OER is = **284.64 x 10^{16}**

Ni-A

Calculated area associated with the reduction peak = 1.35 x 10^{-3} V A

Hence the associated charge is = 1.35 x 10^{-3} V A / 0.005 V s^{-1}

= 270 x 10^{-3} A s

= 270 x 10^{-3} C

Now, the number of electron transferred is = 270 x 10^{-3} C / 1.602 ×10^{-19} C

= 168.53 x 10^{16}

Since the reduction of Ni^{2+} to Ni^{3+} is a single electron transfer reaction, the number of electrons calculated above is the same as the number of surface-active sites.

Hence,

The surface-active site that participated in OER is = **168.53 x 10^{16}**

Co-A

Calculated area associated with the reduction peak = 0.66x 10^{-3} V A

Hence the associated charge is = 0.66 x 10^{-3} V A / 0.005 V s^{-1}

= 132 x 10^{-3} A s

= 132 x 10^{-3} C

Now, the number of electron transferred is = 132 x 10^{-3} C / 1.602 ×10^{-19} C

= 82.39 x 10^{16}

Since the oxidation of Ni^{2+} to Ni^{3+} is a single electron transfer reaction, the number of electrons calculated above is the same as the number of surface-active sites.

Hence,

The surface-active site that participated in OER is = **82.39 x 10^{16}**

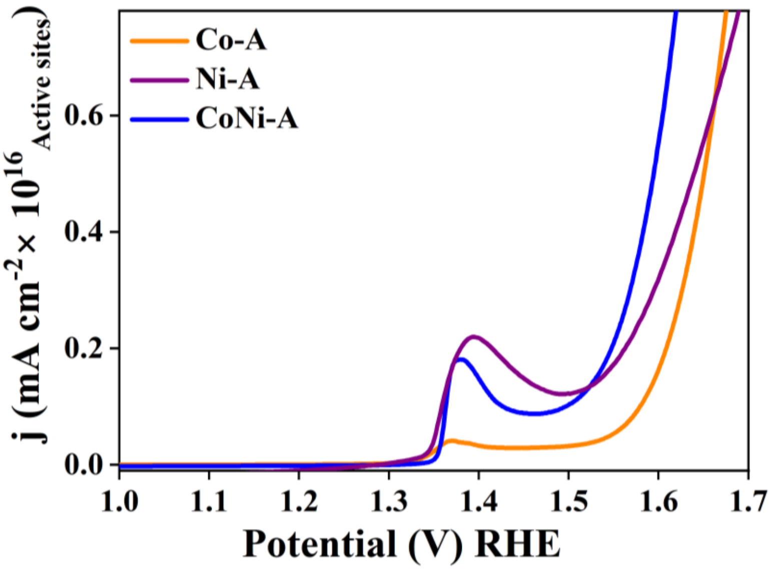

Figure S18. Active site normalized OER activities of the catalysts.

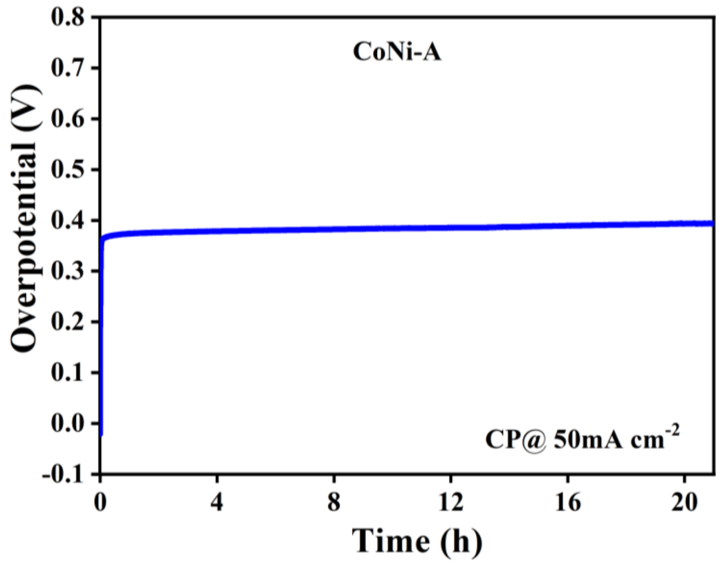

Figure S19. Chronopotentiometry for CoNi-A at 50 mA cm^{-2}.

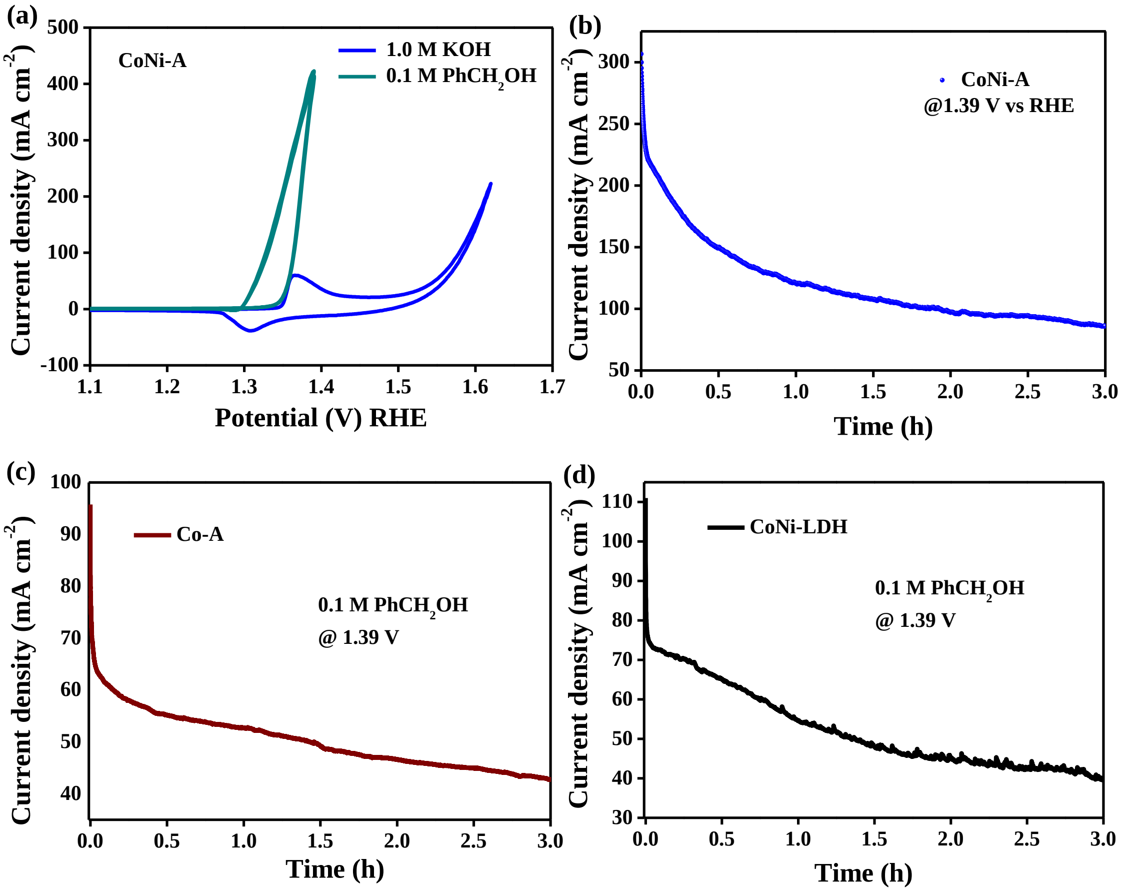

Figure S20. a) CV profiles of CoNi-A in benzyl alcohol demonstrates an increase in current density along with a decrease in required potential; (b-d) Chronoamperometric study of CoNi-A, Co-A, and CoNi-LDH in 0.1 M $PhCH_2OH$ at 1.39 V vs RHE.

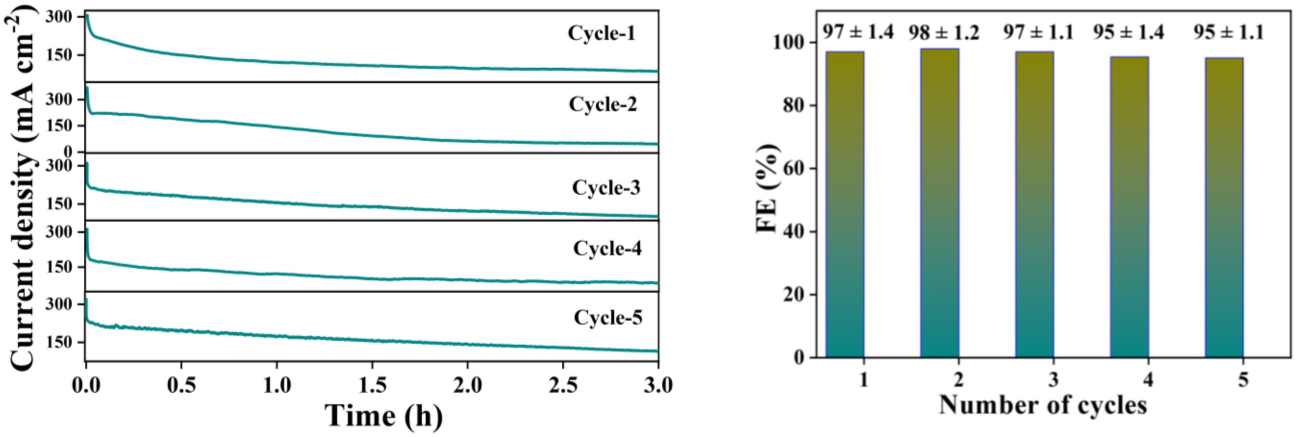

Figure S21. (a) CA graph for five successive cycles for CoNi-A; (b) Plot for the FE measurement of $PhCH_2OH$ oxidation.

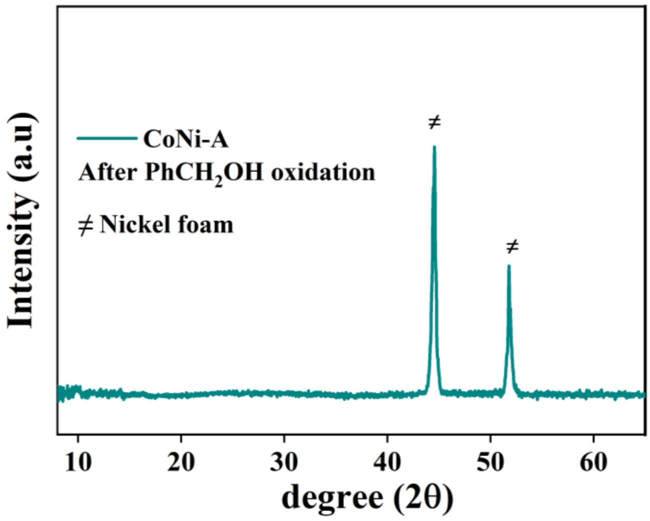

Figure S22. The PXRD pattern of CoNi-A after $PhCH_2OH$ oxidation shows no additional peaks other than those from the nickel foam, indicating the retention of amorphous nature.

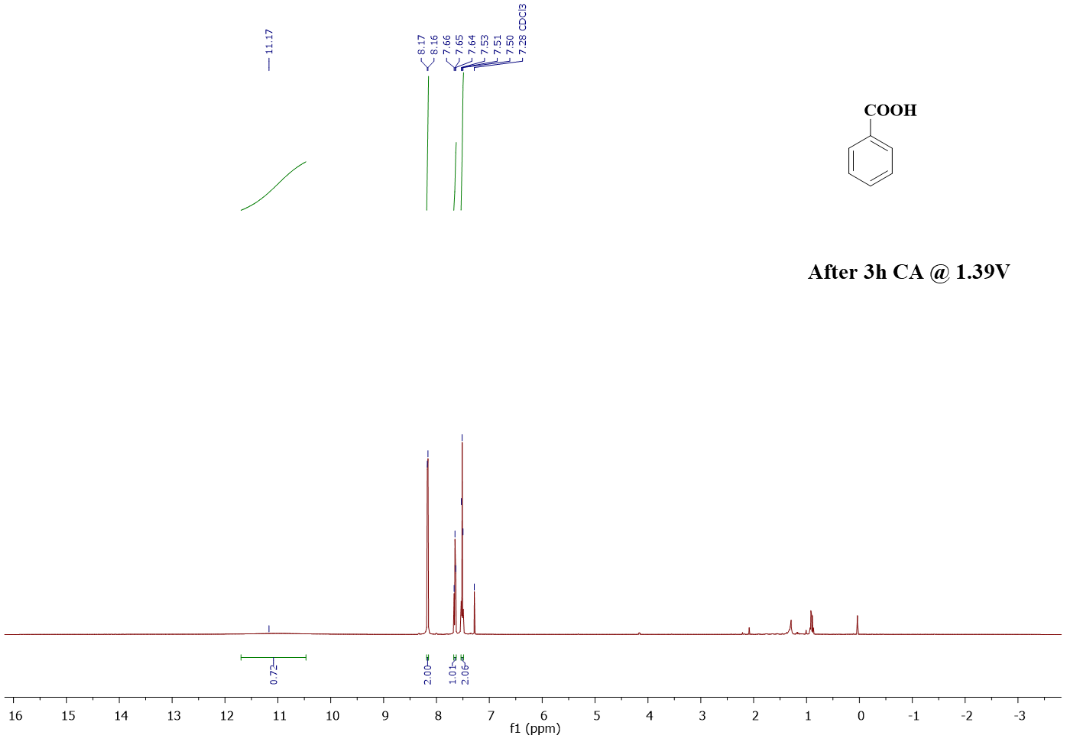

Figure S23. ^{1}H NMR spectrum of benzoic acid formed after 3 h of CA oxidation of 0.1 M benzyl alcohol at 1.39 V with CoNi-A.

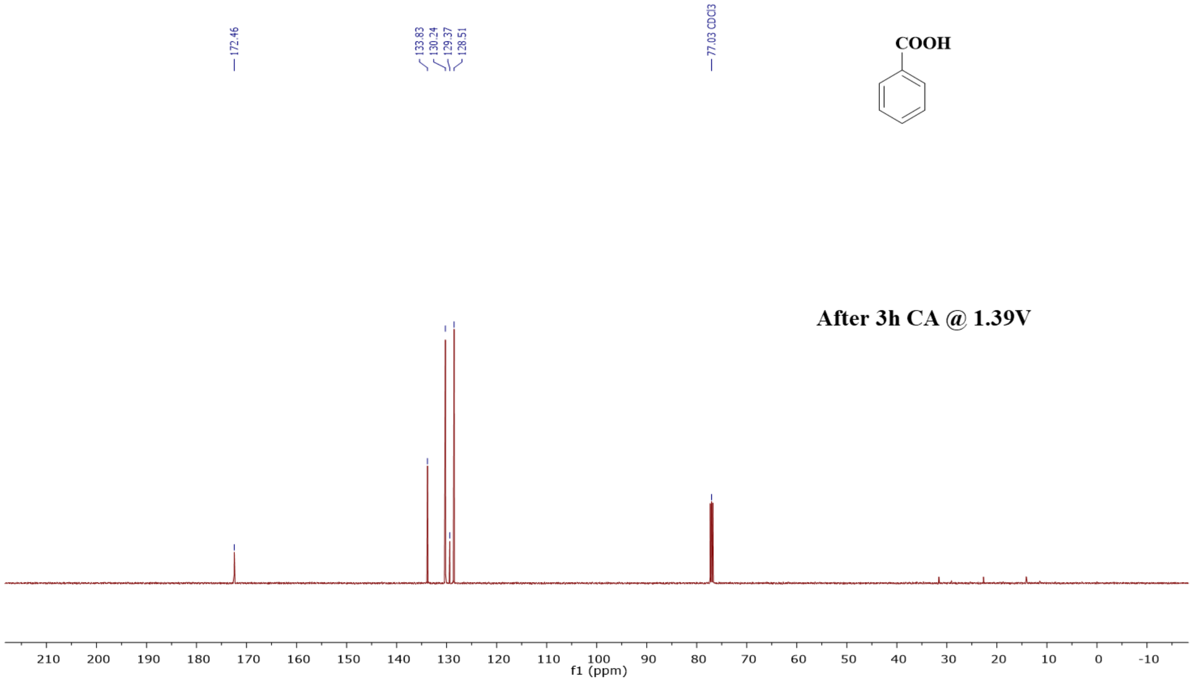

Figure S24. ^{13}C NMR spectrum of benzoic acid formed after 3 h of CA oxidation of 0.1 M benzyl alcohol at 1.39 V with CoNi-A.

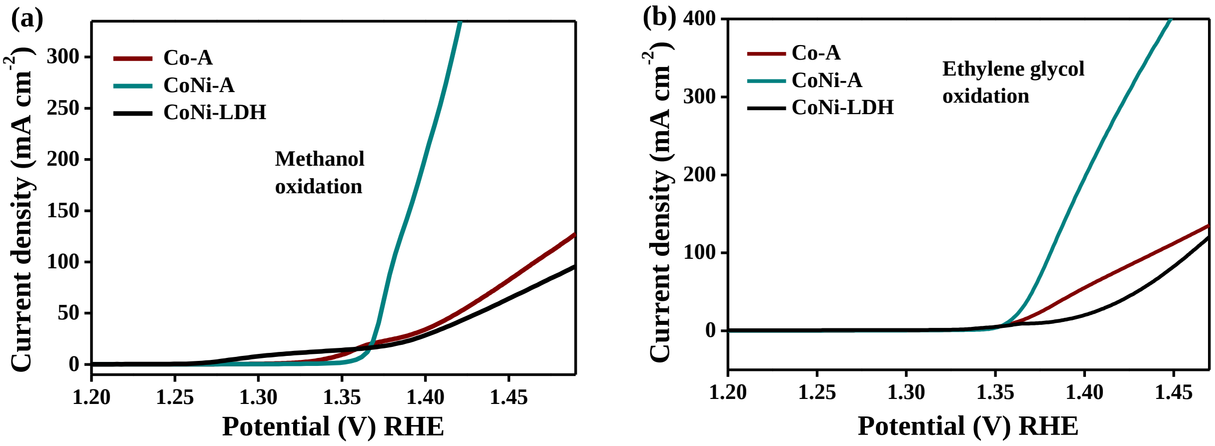

Figure S25. (a) The LSV profiles for methanol oxidation with CoNi-A, Co-A, and CoNi-LDH showing higher activity for CoNi-A. (b) The LSV profiles for ethylene glycol oxidation with CoNi-A, Co-A, and CoNi-LDH exhibiting higher activity for CoNi-A.

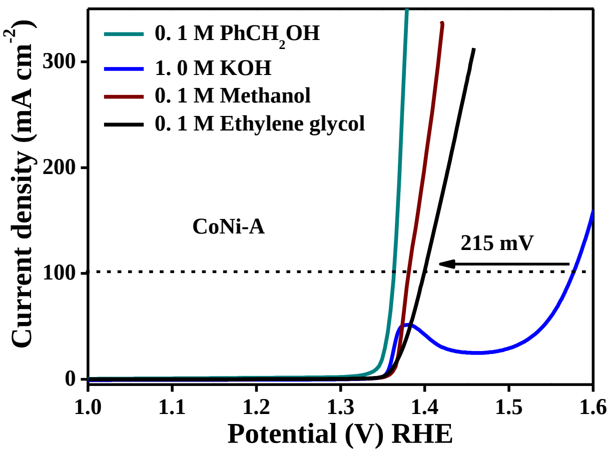

Figure S26. The LSV profiles of methanol, ethylene glycol, and benzyl alcohol oxidation with CoNi-A.

Table 1. Details of the catalyst and their atomic % in EDS analysis

**Precatalyst**	Atomic %	**Active catalyst**	Atomic %
NiCo-PBA	Ni: 20.8, Co: 7.8	CoNi-A	Ni: 14.00, Co: 0.10

Table S2. Comparison of the benzyl alcohol oxidation activity with catalysts CoNi-A and Co-A with literature-reported catalysts.

**Catalyst**	**$PhCH_2OH$ Concentration (M)**	**Current density (mA/cm^2)**	**Potential V vs RHE**	**References**
**Reported catalysts**				
Au/Co(O)OH/NF	0.100	540	1.5	[14]
$Co_{0.83}Ni_{0.17}/C$	0.010	30	1.42	[15]
Co_3O_4 nanowire/NF	0.020	86	1.5	[16]
Co-Ni-LDH/NF	0.010	120	1.6	[17]
NC@$CuCo_2N_x$/CF	0.015	200	1.5	[18]
CoFe-LDH/NF	0.015	50	1.51	[19]
hp-Ni/NF	0.010	10	1.42	[20]
Co/$Ni(OH)_2$/NF	0.100	100	1.33	[21]
A-Ni-Co-H/NF	0.100	100	1.35	[22]
Ni_2P/NF	0.100	30	1.32	[23]
C@NiO/Ni_3S_2	0.200	85	1.42	[24]
$NiCo(OOH)_x$/NF	0.150	50	1.35	[25]
$NiCo_2O_4$/NF	0.050	100	1.46	[26]
N–Mo–Ni/NF	0.100	100	1.33	[27]
Plasma modified NF	0.100	500	1.47	[28]
ZnO/Co_3O_4@$Ni(OH)_2$/NF	0.100	100	1.46	[29]
Fe-Co(O)OH	0.100	400	1.47	[30]
**Present study**				
**CoNi-A**	**0.100 M**	**400**	**1.38**	**This work**
**Co-A**	**0.100 M**	**300**	**1.51**	**This work**
**Ni-A**	**0.100 M**	**300**	**1.48**	**This work**

References

[1] H. Zhang, P. Li, S. Chen, F. Xie, D. J. Riley, *Adv. Funct. Mater.* **2021**, *31*, 2106835.

[2] R. G. Yoshimura, T. V. Thiago, P. J. Zambiazi, J. A. Bonacin, *Energy Adv.* **2023**, *3*, 495–503.

[3] C. Deng, K. H. Wu, X. Lu, S. Cheong, R. D. Tilley, C. L. Chiang, Y. C. Lin, Y. G. Lin, W. Yan, J. Scott, R. Amal, D. W. Wang, *ChemSusChem* **2021**, *14*, 2612–2620.

[4] H. Zhang, P. Li, S. Chen, F. Xie, D. J. Riley, *Adv. Funct. Mater.* **2021**, *31*, 2106835.

[5] X.-Y. Yu, Yi. Feng, B. Guan, X. W. Lou, U. Paik, *Energy Environ. Sci*. **2016**, 9, 1246-1250.

[6] F. Ma, Q. Wu, M. Liu, L. Zheng, F. Tong, Z. Wang, P. Wang, Y. Liu, H. Cheng, Y. Dai, Z. Zheng, Y. Fan, B. Huang, *ACS Appl. Mater. Interfaces* **2021**, *13*, 5142–5152.

[7] B. Singh, P. Mannu, Y. C. Huang, R. Prakash, S. Shen, C. L. Dong, A. Indra, *Angew. Chem. Int. Ed.* **2022**, *134*, 202211585.

[8] B. Singh, T. Ansari, N. Verma, Y. C. Huang, P. Mannu, C. L. Dong, A. Indra, *J Mater. Chem. A* **2024**, DOI 10.1039/d4ta01953a.

[9] J. Du, C. Li, X. Wang, T. G. J. Jones, H. P. Liang, *Electrochim. Acta* **2019**, *303*, 231–238.

[10] Y. Xu, H. Liu, Y. Wu, Q. Wu, C. Li, X. Wang, H. Qin, A. Qin, L. Wang, *ChemNanoMat* **2023**, *9*, 2300414.

[11] Y. Song, X. Wan, Y. Miao, J. Li, Z. Ren, B. Jin, H. Zhou, Z. Li, M. Shao, *Appl. Catal. B* **2023**, *333*, 122808.

[12] G. Liu, X. Zhang, C. Zhao, Q. Xiong, W. Gong, G. Wang, Y. Zhang, H. Zhang, H. Zhao, *New J.Chem*. **2018**, *42*, 6381.

[13] W. Zhang, Y. Dong, M. Huang, Z. Liu, *J. Alloys Compd.* **2023**, *935*,168084.

[14] Z. Li, Y. Yan, S. M. Xu, H. Zhou, M. Xu, L. Ma, M. Shao, X. Kong, B. Wang, L. Zheng, H. Duan, *Nat. Commun.* **2022**, *13*, 147.

[15] G. Liu, X. Zhang, C. Zhao, Q. Xiong, W. Gong, G. Wang, Y. Zhang, H. Zhang, H. Zhao, **2018**, *42*, 6381–6388.

[16] Y. Cao, D. Zhang, X. Kong, F. Zhang, X. Lei, *J. Mater. Sci*. **2021**, *56*, 6689–6703.

[17] N. Shilpa, A. Pandikassala, P. Krishnaraj, P. S. Walko, R. N. Devi, S. Kurungot, *ACS Appl. Mater. Interfaces* **2022**, *14*, 16222–16232.

[18] J. Zheng, X. Chen, X. Zhong, S. Li, T. Liu, G. Zhuang, X. Li, S. Deng, D. Mei, J. G. Wang, *Adv. Funct. Mater* **2017**, *27*,1704169.

[19] Y. Xu, H. Liu, Y. Wu, Q. Wu, C. Li, X. Wang, H. Qin, A. Qin, L. Wang, *ChemNanoMat* **2023**, *9*, 2300414.

[20] B. You, X. Liu, X. Liu, Y. Sun, *ACS Catal.* **2017**, *7*, 4564–4570.

[21] L. Ming, X. Y. Wu, S. S. Wang, W. Wu, C. Z. Lu, *Green Chem.* **2021**, *23*, 7825–7830.

[22] H. Huang, C. Yu, X. Han, H. Huang, Q. Wei, W. Guo, Z. Wang, J. Qiu, *Energy Environ. Sci.* **2020**, *13*, 4990–4999.

[23] F. Li, C. Liu, H. Lin, Y. Sun, H. Yu, S. Xue, J. Cao, X. Jia, S. Chen, *J. Colloid Interface Sci.* **2023**, *640*, 329–337.

[24] R. Li, P. Kuang, L. Wang, H. Tang, J. Yu, *J. Chem. Eng.* **2022**, *431*, 134137.

[25] M. Zhang, Z. Xu, B. Liu, Y. Duan, Z. Zheng, L. Li, Q. Zhou, V. G. Matveeva, Z. Hu, J. Yu, K. Yan, *AIChE J.* **2023**, *69*, 18077.

[26] M. Xu, J. Geng, H. Xu, S. Zhang, H. Zhang, *Inorg. Chem. Front*. **2023**, *10*, 2053–2059.

[27] J. Wan, X. Mu, Y. Jin, J. Zhu, Y. Xiong, T. Li, R. Li, *Green Chem.* **2022**, *24*, 4870–4876.

[28] J. N. Hausmann, P. V. Menezes, G. Vijaykumar, K. Laun, T. Diemant, I. Zebger, T. Jacob, M. Driess, P. W. Menezes, *Adv. Energy Mater.* **2022**, *12*, 202202098.

[29] J. K. Li, A. Wang, X. Y. Dong, S. Huang, Y. Meng, J. L. Song, *New J. Chem.* **2023**, *47*, 5970–5976.

[30] B. Singh, T. Ansari, N. Verma, Y. C. Huang, P. Mannu, C. L. Dong, A. Indra, J. *Mater. Chem. A Mater* **2024**, *12***,** 19321-19330.
